# The storage and recall of memories in the hippocampo-cortical system

**DOI:** 10.1007/s00441-017-2744-3

**Published:** 2017-12-07

**Authors:** Edmund T. Rolls

**Affiliations:** 1 Oxford Centre for Computational Neuroscience, Oxford, England; 2 Department of Computer Science, University of Warwick, Coventry, England

**Keywords:** Completion

## Abstract

A quantitative computational theory of the operation of the hippocampus as an episodic memory system is described. The CA3 system operates as a single attractor or autoassociation network (1) to enable rapid one-trial associations between any spatial location (place in rodents or spatial view in primates) and an object or reward and (2) to provide for completion of the whole memory during recall from any part. The theory is extended to associations between time and object or reward to implement temporal order memory, which is also important in episodic memory. The dentate gyrus performs pattern separation by competitive learning to create sparse representations producing, for example, neurons with place-like fields from entorhinal cortex grid cells. The dentate granule cells generate, by the very small number of mossy fibre connections to CA3, a randomizing pattern separation effect that is important during learning but not recall and that separates out the patterns represented by CA3 firing as being very different from each other. This is optimal for an unstructured episodic memory system in which each memory must be kept distinct from other memories. The direct perforant path input to CA3 is quantitatively appropriate for providing the cue for recall in CA3 but not for learning. The CA1 recodes information from CA3 to set up associatively learned backprojections to the neocortex to allow the subsequent retrieval of information to the neocortex, giving a quantitative account of the large number of hippocampo-neocortical and neocortical-neocortical backprojections. Tests of the theory including hippocampal subregion analyses and hippocampal NMDA receptor knockouts are described and support the theory.

**Electronic supplementary material:**

The online version of this article (10.1007/s00441-017-2744-3) contains supplementary material, which is available to authorized users.

## Introduction

A computational theory of the operation of networks in the hippocampus in memory (Kesner and Rolls 2015; Rolls 2010, 2016a) is described. The type of memory is episodic, referring to the memory of a particular event or linked group of events occurring typically at the same time and place. An example might be where dinner was yesterday, who was present, who sat where, what the menu was and the discussion. This must be kept separate from, for example, what happened the day before that. Episodic memory almost always has a spatial component (Dere et al. 2008; Rolls 2017) and a spatial representation in the hippocampus can be updated by self-motion to produce path integration (McNaughton et al. 1996; Robertson et al. 1998; Rolls 2016a; E.T. Rolls and S. Wirth in preparation).

Episodic memory can be operationally investigated in animals including humans in the following ways. First is the ability to store rapidly, on a single trial, a unique combination of inputs that typically involve place or time and objects including people and, later, to recall the whole memory from any part. The episodic memory, in being formed rapidly, is relatively unstructured and may be formed simply by associating together the spatial or temporal and object representations. In contrast, a semantic memory has structure and may require many exemplars to learn the representation, as exceptions might occur, such as that an ostrich is a bird but cannot fly (McClelland et al. 1995). The recall of many episodic memories from the hippocampus may help to build semantic memories in the neocortex, for example, a map of the world based on the journeys that one has made. An example of a semantic representation is a Jennifer Aniston neuron, which may respond not only to Jennifer Aniston but also to other actors in the same movie and the places with which they are associated (Quiroga 2012; Rey et al. 2015). These neurons are probably formed in high-order neocortical areas in the temporal lobes and their junction with the parietal lobes; and their presence in the medial temporal lobe (Quiroga 2012), for example, the parahippocampal gyrus, is probably because the hippocampal system receives input from these high-order neocortical areas. Autobiographical memory is a semantic memory that involves representations of the self, frequently involving the precuneus (Bubb et al. 2017; Cavanna and Trimble 2006; Fossati 2013) and that might be built by using the recall of episodic memories. A second property of an episodic memory is that it may involve a temporal sequence of events. The hippocampus has mechanisms that help to implement this (Eichenbaum 2014; Howard and Eichenbaum 2015; Kesner and Rolls 2015; Kraus et al. 2013b), as described below.

The theory of the hippocampus and episodic memory is based on the remarkable neural architecture of the hippocampus, on the effects of damage to it and on the neuronal activity recorded in it. Once memories have been stored in the hippocampus, they may later need to be recalled to the neocortex; a theory of the recall mechanism is part of the overall theory (Treves and Rolls 1994; see also Backprojections to the neocortex and memory recall). Once recalled to the neocortex, the memories of particular events or episodes can be reported verbally and, hence, this is a type of declarative memory (Squire and Wixted 2011). The recalled information may also be combined with other information to be reorganized and stored semantically in the neocortex, i.e., in a form that reflects meaning and structure, in contrast to the episodic memories captured as discrete memories by the hippocampus (McClelland et al. 1995). An example of a semantic representation might be a mental map that includes and describes the relationships between the places to which one has made particular journeys. I start with a description of the underlying architecture and functions of the hippocampus in order to provide a firm foundation for the theory and then show ways in which the theory is being tested experimentally.

## Overview

Some of the key points in the computational theory are as follows. The hippocampal CA3 system operates as a single attractor or autoassociation network (1) to enable rapid one-trial associations between any spatial location (place in rodents or spatial view in primates) and an object or reward and (2) to provide for completion of the whole memory during recall from any part. The theory is extended to associations between time and object or reward to implement temporal order memory, which is also important in episodic memory. The dentate gyrus performs pattern separation by competitive learning to produce sparse representations, producing, for example, neurons with place-like fields from entorhinal cortex grid cells. The dentate granule cells produce, by the very small number of mossy fibre connections to CA3, a randomizing pattern separation effect that is important during learning but not recall and that separates out the patterns represented by CA3 firing as being very different from each other; this is optimal for an unstructured episodic memory system in which each memory must be kept distinct from other memories. The direct perforant path input to CA3 projection is quantitatively appropriate to provide, as a pattern association mechanism, the cue for recall in CA3. The CA1 recodes information from CA3 in order to set up associatively learned backprojections to the neocortex to allow the subsequent retrieval of information to the neocortex, providing a quantitative account of the large number of hippocampo-neocortical and neocortical-neocortical backprojections. Empirical tests of the theory including hippocampal subregion analyses and selective hippocampal NMDA receptor knockouts are described and support the theory.

## Structure and function of the hippocampal system

### Effects of damage to the hippocampus

In the patient H.M., bilateral damage to the hippocampus performed to treat epilepsy produced an inability to remember “recent” events (those since the hippocampal and related damage), while leaving the memory of events that occurred prior to the hippocampal damage and semantic and skill memory relatively unimpaired (Corkin 2002; Scoville and Milner 1957). In tests to examine the exact brain regions that impair this memory for events, tasks that require objects to be associated with the place in which they are located have been shown to be especially sensitive to hippocampal damage. Examples include memory for the location of an escape platform in a water bath in rats (Andersen et al. 2007; Morris and Frey 1997) and for the location of an odour signifying the place where a food will be found in a cheeseboard task (Kesner and Rolls 2015). Temporal order memory for a sequence of places or objects is also impaired by hippocampal damage (Kesner and Rolls 2015) and this functionality may be important in temporally linking a sequence of events within an episodic memory. In monkeys, analogous tasks involving object-place memory are impaired by hippocampal damage (Banta Lavenex and Lavenex 2009), whereas damage to the overlying perirhinal cortex, which is connected to the inferior temporal cortex system involved in the computation of invariant object representations (Rolls 2012c, 2016a), impairs a different type of memory more closely related to perceptual functions, namely recognition memory for objects (Buckley 2005).

These deficits produced by hippocampal damage underlie the importance of the hippocampus in event memory in which there is frequently an association of a place with an object. This provides a prototypical paradigm in which to analyse and conceptualize hippocampal computation.

### Systems-level anatomy

**Fig. 1:**
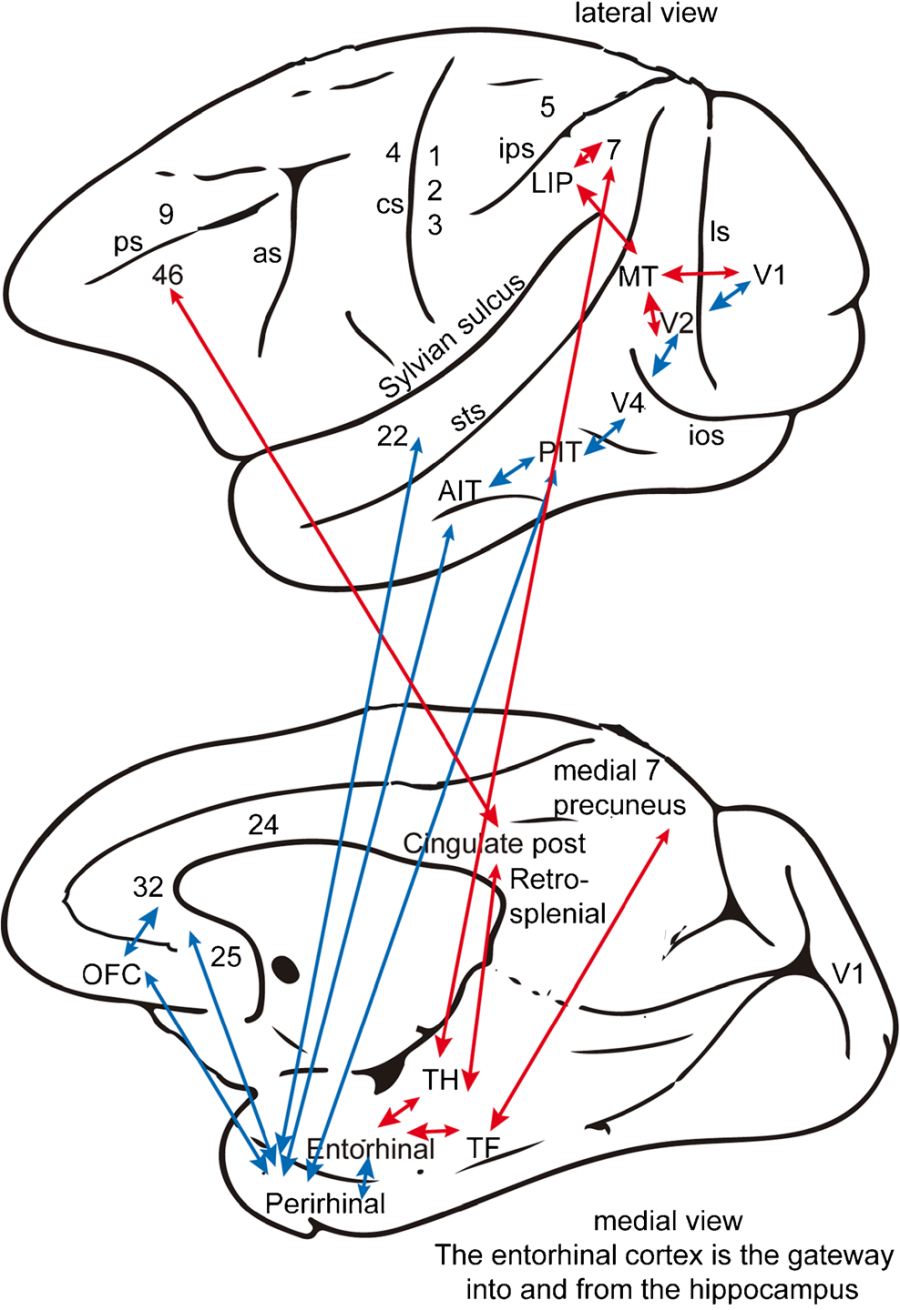
Connections of the primate hippocampus with the neocortex. Macaque brain. *Top* Lateral view. *Bottom* Medial view. The hippocampus receives its inputs via the parahippocampal gyrus (areas *TF* and *TH*) and the perirhinal cortex (areas 35 and 36), both of which in turn project to the entorhinal cortex (area 28), send inputs to the hippocampus and receive backprojections from the hippocampus, as shown in Fig. 2. The forward inputs towards the entorhinal cortex and hippocampus are shown with *large arrowheads* and the weaker return backprojections with *small arroweads*. The hippocampus receives input via the perirhinal cortex areas 35 and 36, which project to the lateral entorhinal cortex areas 28 from the ends of the hierarchically organized ventral visual system pathways (*V1*, *V2*, *V4*, *PIT*, *AIT*) that represent “what” object is present (including faces and even scenes), from the anterior inferior temporal visual cortex (*AIT*, BA21, TE) where objects and faces are represented and that receives input from the posterior inferior temporal cortex (*PIT*, BA20, TEO), from the reward system in the orbitofrontal cortex (*OFC*) and amygdala, from an area to which the OFC projects, namely the anterior cingulate cortex BA32 and subgenual cingulate cortex (BA25), from the high-order auditory cortex (BA22) and from olfactory, taste and somatosensory “what” areas (not shown). These ventral “what” pathways are shown in *blue*. The hippocampus also receives via the parahippocampal cortex areas TF and TH inputs (shown in *red*) from the dorsal visual “where” or “action” pathways, which reach parietal cortex area 7 via the dorsal visual stream hierarchy, including V1, V2, MT, MST, LIP and VIP and from areas to which they are connected, including the dorsolateral prefrontal cortex BA46 and the posterior cingulate and retrosplenial cortex (*as* arcuate sulcus, *cs* central sulcus, *ips* intraparietal sulcus, *ios* inferior occipital sulcus, *ls* lunate sulcus, *sts* superior temporal sulcus). The hippocampus provides a system for all the high-order cortical regions to converge into a single network in the hippocampal CA3 region, as shown in Fig. 2 (Rolls 2015b, 2016a)

**Fig. 2:**
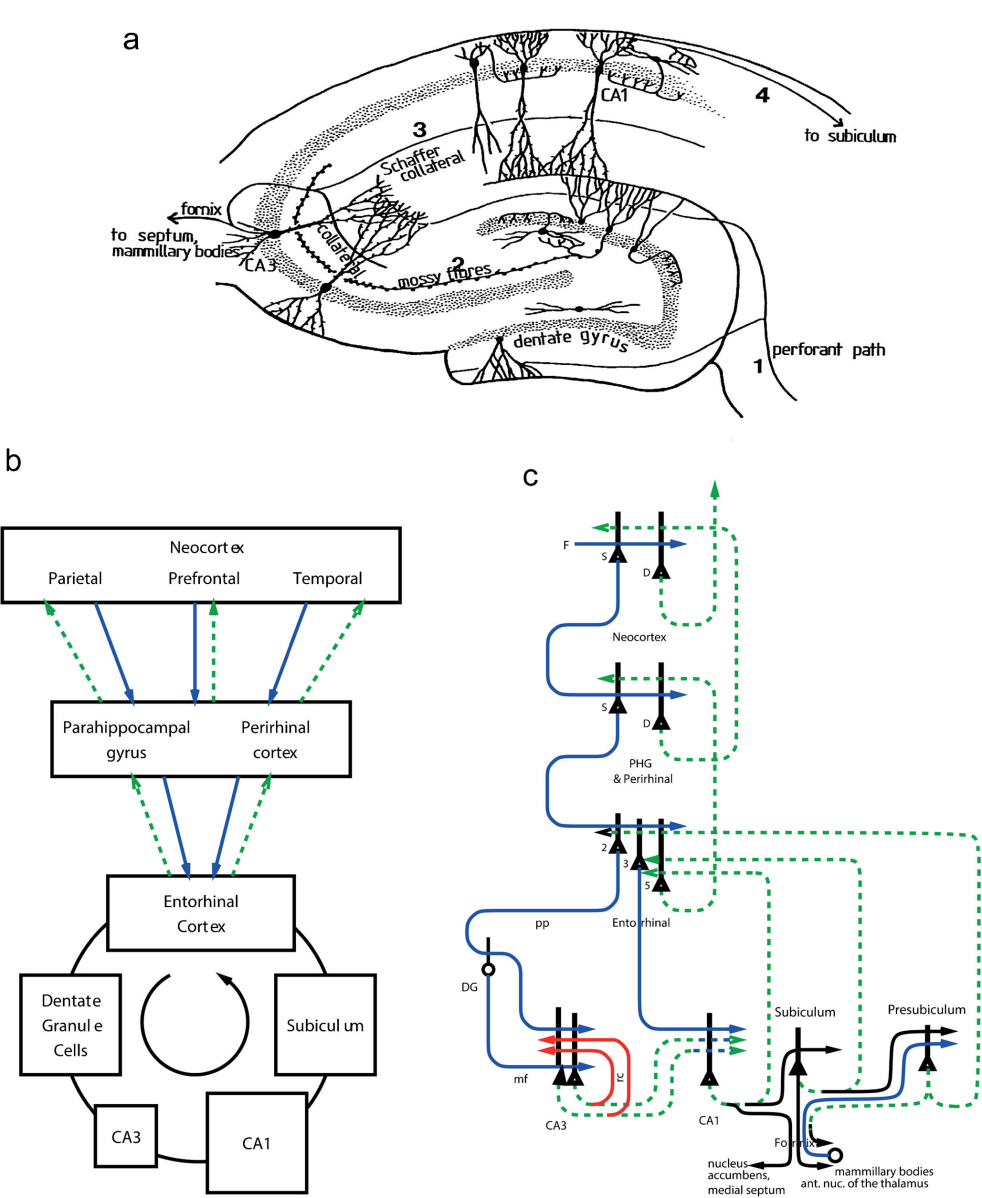
**a** Representation of connections within the hippocampus. Inputs reach the hippocampus through the perforant path (*1*), which makes synapses with the dendrites of the dentate granule cells and also with the apical dendrites of the CA3 pyramidal cells. The dentate granule cells project via the mossy fibres (*2*) to the CA3 pyramidal cells. The well-developed recurrent collateral system of the CA3 cells is indicated. The CA3 pyramidal cells project via the Schaffer collaterals (*3*) to the CA1 pyramidal cells, which in turn have connections (*4*) to the subiculum. **b**, **c** Forward connections (*solid blue lines*) from areas of the cerebral association neocortex via the parahippocampal gyrus and perirhinal cortex and entorhinal cortex to the hippocampus and backprojections (*dashed green lines*) via the hippocampal CA1 pyramidal cells, subiculum and parahippocampal gyrus to the neocortex. Great convergence occurs in the forward connections down to the single network implemented in the CA3 pyramidal cells and great divergence again in the backprojections. **b** Block diagram. **c** More detailed representation of some of the principal excitatory neurons in the pathways. The CA3 recurrent collateral connections are shown in *red* (*D* deep pyramidal cells, *DG* dentate granule cells, *F* forward inputs to areas of the association cortex from preceding cortical areas in the hierarchy, *mf* mossy fibres, *PHG* parahippocampal gyrus and perirhinal cortex, *pp* perforant path, *rc* recurrent collateral of the CA3 hippocampal pyramidal cells, *S* superficial pyramidal cells, *2* pyramidal cells in layer 2 of the entorhinal cortex, *3* pyramidal cells in layer 3 of the entorhinal cortex). The *thick lines above* the cell bodies represent dendrites

To understand the functions of the primate hippocampus in event or episodic memory, we need to understand from which other parts of the brain it receives information and to what it in turn connects. The primate hippocampus receives inputs via the entorhinal cortex (Brodmann area 28), via the highly developed parahippocampal gyrus (areas TF and TH) and via the perirhinal cortex from the ends of many processing streams of the cerebral association cortex, including the visual and auditory temporal lobe association cortical areas, the prefrontal cortex and the parietal cortex (Lavenex and Amaral 2000; Lavenex et al. 2004; Rolls 2016a; E.T. Rolls and S. Wirth in preparation; Suzuki and Amaral 1994b; Van Hoesen 1982; van Strien et al. 2009; see Figs. 1, 2b). The hippocampus is thus, by its connections, potentially able to associate together object and spatial representations. In addition, the entorhinal cortex receives inputs from the amygdala and the orbitofrontal cortex, which could provide reward/valence-related information to the hippocampus (Carmichael and Price 1995; Pitkanen et al. 2002).

The primary output from the hippocampus to neocortex originates in CA1 and projects to the subiculum, entorhinal cortex and parahippocampal structures (areas TF-TH) and to the prefrontal cortex (Delatour and Witter 2002; van Haeften et al. 2003; Van Hoesen 1982; van Strien et al. 2009; see Figs. 1, 2b), although other outputs have been found (Kesner and Rolls 2015). These are the pathways that are likely to be involved in the recall of information from the hippocampus back to the rest of the neocortex.

### Neurophysiology of the hippocampus

The systems-level neurophysiology of the hippocampus shows the information that could be stored or processed by the hippocampus. To understand the way that the hippocampus works, we need to state more than just that it can store information - one needs to know what information.

#### Rodent place cells

In rodents, place cells, which respond when a rat is near a particular place, are found in the hippocampus (Hartley et al. 2014; Jeffery 2011; McNaughton et al. 1983; O’Keefe and Dostrovsky 1971). Place cells are found in regions CA3 and CA1 (with smaller place fields in the dentate granule cells; Neunuebel and Knierim 2012; see Fig. 2a for the architecture of the hippocampus). The representation is allocentric (as contrasted with egocentric) in that the neurons fire whenever the rat is in the place field, typically independently of the head direction of the rat. In the medial entorhinal cortex, grid cells are present that have regularly spaced peaks of firing in an environment, so that as a rat runs through an environment, a single neuron increases then decreases its firing a number of times as the rat traverses the environment (Moser et al. 2015; see also below). The grid cell system appears to provide ring continuous attractors that would be useful not only for spatial path integration (computing position based on self-motion; Giocomo et al. 2011; McNaughton et al. 2006) but also for the timing information during sequence encoding for non-spatial and spatial information (Kesner and Rolls 2015), as described in the section Entorhinal cortex grid cells.

#### Primate spatial view cells and object-spatial view cells

**Fig. 3:**
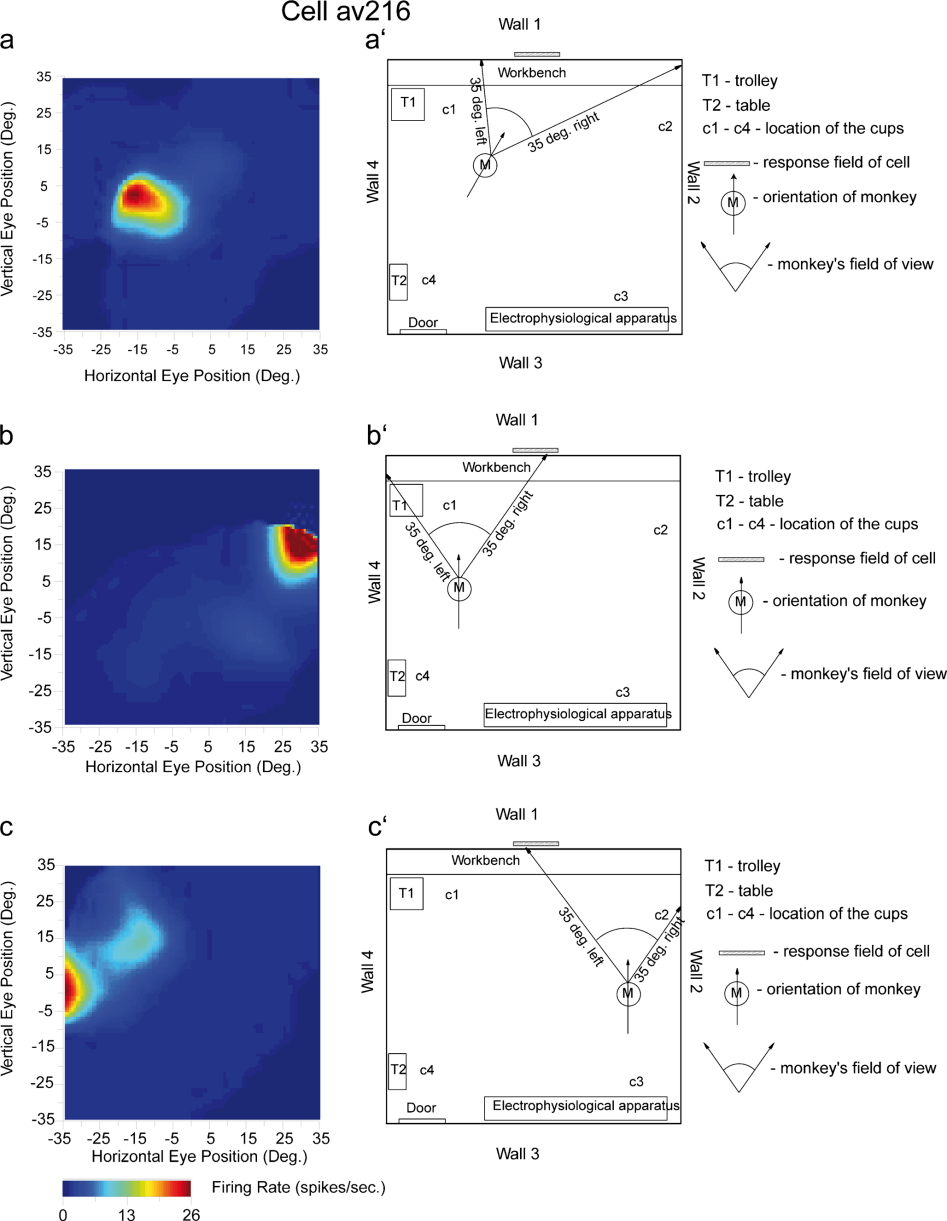
Examples of the firing of a hippocampal spatial view cell (*av216*) when the monkey was at various positions in the room, with various head directions, looking at wall 1 of the room. The details of the spatial view field are shown by the different firing rates with the *colour calibration bar* shown *below*. The firing rate of the cell in spikes/s as a function of horizontal and vertical eye position is indicated by the colour in each diagram *left* (with the *calibration bar* in spikes/s shown *below*). Positive values of eye position represent *right* in the horizontal plane and *up* in the vertical plane (*hatched box right* approximate position of spatial view field). The diagram provides evidence that the spatial view field is in allocentric room-based coordinates and not eye position or place coordinates (for details see Georges-François et al. 1999)

In monkeys, which are used as a model to help understand human memory, there is a prominent representation of spatial view, the location at which the primate is looking (E.T. Rolls and S. Wirth in preparation; Rolls and Xiang 2006; for example, see Fig. 3). The representation of spatial view is allocentric, in that it is independent of the place at which the monkey is located in the room or of the eye position in the head (left gaze vs right gaze) and of the head direction but depends on the location in space being viewed (Georges-François et al. 1999). The spatial view cells are updated by self-motion (e.g., moving the eyes or running to a new part of the environment) indicating that path integration is implemented (Robertson et al. 1998). This type of representation is much more appropriate for a human memory system than that in rodents, because a human can remember where an object or person has been seen based just on looking at the place, without necessarily ever having been at that place (Rolls 2017); for example, you may remember where you have seen a lecturer in a lecture theatre, without ever having visited the precise place where the lecturer is standing. Moreover, some spatial view neurons respond to particular combinations of object and place, such as that object 1 is in place 1 on a screen (Rolls et al. 2005) or that reward 1 is at place 1 on a screen (Rolls and Xiang 2005) and thus seem to encode what is necessary for an object-place memory representation system in the brain (Rolls 2016a, 2017; Rolls and Xiang 2006). Further, some of these neurons reflect the completion during recall of a whole memory from a part, for example, of the spatial location at which an object has previously been shown (Rolls and Xiang 2006). Consistent with these findings and with the computational theory, human hippocampal neurons have now been reported to be activated during recall (Gelbard-Sagiv et al. 2008). View cells modulated by place have been found in monkeys (Rolls and O’Mara 1995; E.T. Rolls and S. Wirth in preparation; Wirth et al. 2017) and great care is needed to establish, by eye position recordings taken together with recordings of the place where the monkey is located during locomotion (Georges-François et al. 1999; Robertson et al. 1998; Rolls 1999; Rolls et al. 1997a, 1998), that there is information about place as well as about spatial view in primates (E.T. Rolls and S. Wirth in preparation). Evidence consistent with the presence of spatial view cells in the primate hippocampus is that spatial view grid cells have been described in the monkey entorhinal cortex (Buffalo 2015; Killian et al. 2012; Rueckemann and Buffalo 2017). These neurons correspond to place-related grid cells in rodents but, in primates, the grid is for the space being looked at, instead. In humans, places being viewed on a video monitor (i.e., spatial views being looked at), not places where the human is actually located, can activate hippocampal neurons (Ekstrom et al. 2003).

Results consistent with object-place neurons in primates (Rolls and Xiang 2006; Rolls et al. 2005) are that some hippocampal neurons in rats respond on the basis of the conjunction of location and odour (Wood et al. 1999). Further, Diamond and colleagues showed, by using the vibrissa somatosensory input for the “object” system, that rat hippocampal neurons respond to object-place combinations, objects or places (Itskov et al. 2011).

#### Reward-related inputs to the hippocampus

The primate anterior hippocampus (which corresponds to the rodent ventral hippocampus) receives inputs from brain regions involved in reward processing such as the amygdala and orbitofrontal cortex (Carmichael and Price 1995; Pitkanen et al. 2002; Stefanacci et al. 1996; Suzuki and Amaral 1994a). To investigate how this affective input is incorporated into primate hippocampal function, Rolls and Xiang (2005) recorded neuronal activity while macaques performed a reward-place association task in which each spatial scene shown on a video monitor had one location that, if touched, yielded a preferred fruit juice reward and a second location that yielded a less preferred juice reward. Each scene had different locations for the different rewards. Of 312 hippocampal neurons analysed, 18% responded more to the location of the preferred reward in different scenes and 5% to the location of the less preferred reward (Rolls and Xiang 2005). When the locations of the preferred rewards in the scenes were reversed, 60% of 44 neurons tested reversed the location to which they responded, showing that the reward-place associations could be altered by new learning in a few trials. The majority (82%) of these 44 hippocampal reward-place neurons tested did not respond to object-reward associations in a visual discrimination object-reward association task. Thus, the primate hippocampus contains a representation of the reward associations of places “out there” being viewed; this is a way in which affective information can be stored as part of an episodic memory and in which the current mood state can influence the retrieval of episodic memories. Consistent evidence has been presented showing that rewards available in a spatial environment can influence the responsiveness of rodent place neurons (Hölscher et al. 2003; Redila et al. 2014; Tabuchi et al. 2003). Further evidence that reward-related information reaches the primate hippocampus is that when macaques learn a novel object-place task, some hippocampal neurons respond to correct outcomes, and others to error outcomes (Wirth et al. 2009).

In humans, reward and non-reward information reaches the hippocampus and appears to be related to the ruminating sad memories present in depression. In depression, there is reduced functional connectivity of the medial orbitofrontal cortex reward-related system with the parahippocampal gyrus and increased functional connectivity of the lateral orbitofrontal cortex non-reward related system, which is implicated in depression (Rolls 2016b), with the precuneus (W. Cheng et al. in preparation) and posterior cingulate cortex (W. Cheng et al. in preparation), areas that provide access to the hippocampal system and that are involved in representations of space and of the self (Cavanna and Trimble 2006; Rolls 2015b).

### Internal structure and connectivity of the hippocampus

The internal hippocampal circuitry is shown in Fig. 2a (Amaral et al. 1990; Amaral and Witter 1989; Andersen et al. 2007; Kondo et al. 2009; van Strien et al. 2009). Projections from the entorhinal cortex layer 2 reach the granule cells (of which there are 10^6^ in the rat) in the dentate gyrus (DG), via the perforant path (pp; Witter 1993). The granule cells project to CA3 cells via the mossy fibres (mf), which provide a sparse but possibly powerful connection to the 3^.^10^5^ CA3 pyramidal cells in the rat. Each CA3 cell receives approximately 46 mossy fibre inputs, so that the sparseness of this connectivity is thus 0.005%. By contrast, there are also many more, possibly weaker, direct perforant path inputs from layer 2 of the entorhinal cortex onto each CA3 cell: in the rat, of the order of 4^.^10^3^. The largest number of synapses (about 1.2^.^10^4^ in the rat) on the dendrites of CA3 pyramidal cells is, however, provided by the (recurrent) axon collaterals of CA3 cells themselves (rc; see Fig. 3). Remarkably, the recurrent collaterals are distributed to other CA3 cells largely throughout the hippocampus (Amaral et al. 1990; Amaral and Witter 1989, 1995; Ishizuka et al. 1990; Witter 2007), so that effectively the CA3 system provides a single network with a connectivity of approximately 2% between the different CA3 neurons given that the connections are bilateral. The CA3-CA3 recurrent collateral system is even more extensive in macaques than in rats (Kondo et al. 2009). The neurons that comprise CA3, in turn, project to CA1 neurons via the Schaffer collaterals. In addition, projections that terminate in the CA1 region originate in layer 3 of the entorhinal cortex (see Fig. 2b; van Strien et al. 2009).

## Theory of the operation of hippocampal circuitry as a memory system

### Introductory remarks

In this section, I consider the way that an event or episodic memories might be stored in and retrieved by hippocampal circuitry and in addition retrieved back into the neocortex where they may be incorporated into long-term semantic or autobiographical memory (Kesner and Rolls 2015). The theory has been developed through many stages (Rolls 1987, 1989b; Rolls 1995; Treves and Rolls 1992, 1994) with fuller accounts and recent developments available (Kesner and Rolls 2015; Rolls 2016a). The theory illustrates the importance of taking into account the details of the circuitry involved in the development of theories of brain function. Some background is that many of the synapses in the hippocampus show associative modification, as revealed by long-term potentiation and that this synaptic modification appears to be involved in learning (Andersen et al. 2007; Takeuchi et al. 2014; Wang and Morris 2010). Early work by David Marr (1971) showed the manner in which associatively modified recurrent connectivity could support pattern completion but he did not identify the CA3 network of the hippocampus as being the crucial network with an appropriate architecture for this to occur. This type of network became known as an autoassociation network (because a pattern is associated with itself by using the recurrent collaterals) in further work by Kohonen (1977) and also became known as an attractor network following the quantitative approach in which partial patterns could be attracted into a basin of attraction (Amit 1989; Hopfield 1982).

A description of the operation of autoassociation networks is provided elsewhere (Amit 1989; Hertz et al. 1991; Rolls and Treves 1998) including *Cerebral Cortex: Principles of Operation* (Rolls 2016a), the Appendices of which are online at www.oxcns.org and a summary is provided in Supplementary Material Box 1, with illustrative simulations available as exercises (Rolls 2016a).

### CA3 as an autoassociation or attractor memory

#### Arbitrary associations and pattern completion in recall

**Fig. 4:**
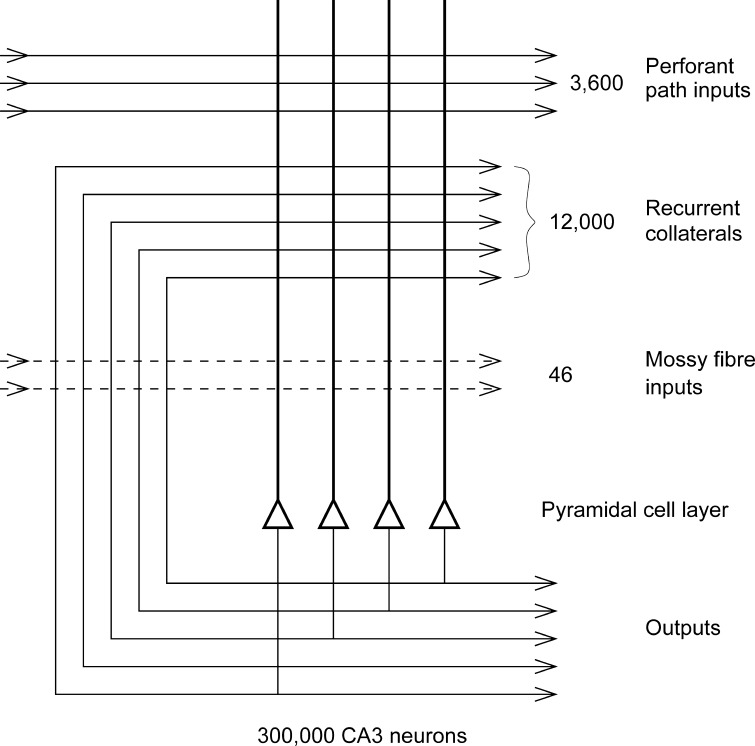
Numbers of connections from three different sources onto each CA3 cell in the rat. After Rolls and Treves (1998) and Treves and Rolls (1992)

On the basis of the evidence summarized above concerning the connectivity of the hippocampus, on the autoassociation networks and on the role of synaptic modification in memory, Rolls (1987, 1989b) at the Dahlem Conference in 1985 on The Neural and Molecular Bases of Learning (Rolls 1987) and others (Levy 1989; McNaughton 1991; McNaughton and Morris 1987) suggested that the CA3 stage acts as an autoassociation memory that enables episodic memories to be formed and stored in the CA3 network and that, subsequently, the extensive recurrent collateral connectivity allows for the retrieval of a whole representation to be initiated by the activation of some small part of the same representation (the cue). The crucial synaptic modification for this is in the CA3 recurrent collateral synapses (see Figs. 1, 2, 4).

The hypothesis is that because the CA3 operates effectively as a single network, it can allow arbitrary associations between inputs originating from very different parts of the cerebral cortex to be formed. These might involve associations between information originating in the temporal lobe visual cortex about the presence of an object (Rolls 2012c) and information originating from scene-selective representations about where the object is (Kornblith et al. 2013; Nasr et al. 2011). Each event might be stored in as little as 1 s (because autoassociation memories require only one-shot learning and long-term potentiation needs only brief inputs) and each event would consist of the vector of neurons firing at the inputs to the hippocampus. An episodic memory might be a single event or a sequence of events. Although some spatial gradient is present in the CA3 recurrent connections, so that the connectivity is not fully uniform in the rat (Ishizuka et al. 1990; Witter 2007) with, very interestingly, there being less gradient in primates (Kondo et al. 2009), the network will still have the properties of a single interconnected autoassociation network allowing associations between arbitrary neurons to be formed, given the presence of many long-range connections that overlap from different CA3 cells and given the ability of attractor networks to operate with diluted connectivity shown in our computational studies prompted by this problem (Treves 1990; Treves and Rolls 1991). The connectivity of the CA3 network is diluted (as contrasted with fully connected) in that there are only approximately 12,000 recurrent collateral synapses on each CA3 neuron and 300,000 CA3 neurons (as shown in Fig. 4). Indeed, diluted connectivity in autoassociation networks in the brain, for example, those in CA3 and those implemented by recurrent collaterals in the neocortex, have been suggested to be advantageous by reducing the probability of multiple connections between any pair of neurons; such multiple connections, if present, would distort the basins of attraction and considerably reduce the number of memories that can be stored (Rolls 2012a, 2016a). The diluted connectivity is also advantageous in the pattern association and competitive networks in the hippocampal system (Rolls 2015a, 2016a, c; Rolls and Webb 2012).

Crucial issues include: how many memories can be stored in this system (to determine whether the autoassociation hypothesis leads to a realistic estimate of such number of memories that the hippocampus can store); whether the whole of a memory can be completed from any part; whether the autoassociation memory can act as a short-term memory, for which the architecture is inherently suited; whether the system can operate with spatial representations that are essentially continuous because of the continuous nature of space; and when the system stores or recalls information. These and related issues are considered below and in more detail elsewhere (Kesner and Rolls 2015; Rolls 2010, 2013a, 2013b, 2016a).

#### Storage capacity

We performed quantitative analyses of the storage and retrieval processes in the CA3 network (Treves and Rolls 1991, 1992). We extended previous formal models of autoassociative memory (Amit 1989; Hopfield 1982) by analysing a network with graded response units, so as to represent more realistically the continuously variable rates at which neurons fire and with incomplete connectivity (Rolls 2016a; Rolls and Treves 1998; Rolls et al. 1997b; Treves 1990; Treves and Rolls 1991). We found that, in general, the maximum number *p*_max_ of firing patterns that can be (individually) retrieved is proportional to the number *C* ^RC^ of (associatively) modifiable recurrent collateral synapses on to each neuron, by a factor that increases roughly with the inverse of the sparseness *a* of the neuronal representation (defined below). Each memory is precisely defined in the theory: it is a set of firing rates of the population of neurons (which represent a memory) that can be stored and later retrieved, with retrieval being possible from a fraction of the originally stored set of neuronal firing rates (Rolls 2016a). The neuronal population sparseness *a* of the representation can be measured by extending the binary notion of the proportion of neurons that are firing to any one stimulus or event as:

1 $$ a=\left({\sum}_{i=1,N}{r}_i/N\right){}^2/{\sum}_{i=1,N}\left({r_i}^2/N\right) $$

where *r*_*i*_ is the firing rate (e.g., spikes/s, typically in the range 0–100 spikes/s) of the *i*’th neuron in the set of *N* neurons. The sparseness ranges from 1/*N*, when only one of the neurons responds to a particular stimulus (a local or grandmother cell representation; Rolls and Treves 2011), to a value of 1.0, attained when all the neurons are responding at the same rate to a given stimulus (Franco et al. 2007; Rolls and Treves 2011; Treves and Rolls 1991). The maximum number of patterns, *p*_max_, that can be stored and correctly retrieved is approximately:

2 $$ {p}_{\mathrm{max}}\cong \frac{C^{\mathrm{RC}}}{a\ln \left(1/a\right)}k $$

where *C* ^RC^ is the number of recurrent collateral connections onto each neuron and *k* is a scaling factor that depends weakly on the detailed structure of the rate distribution, on the connectivity pattern, etc., but that is roughly in the order of 0.2–0.3 (Treves and Rolls 1991). For example, for *C* ^RC^ = 12,000 associatively modifiable recurrent collateral synapses onto each neuron and *a* = 0.02, *p*_max_ is calculated to be approximately 36,000. This analysis emphasizes the utility of having a sparse representation in the hippocampus, for sparse distributed representations increase the number of different memories that can be stored (Treves and Rolls 1991), a feature that is essential for an episodic memory (Rolls 2010, 2016a).

In order for most associative networks to store information efficiently, both heterosynaptic Long Term Depression (LTD) in which a synapse decreases in strength when the presynaptic term is low and the postsynaptic term is high and Long Term Potentiation (LTP) in which an increase occurs in synaptic strength when both the pre- and post-synaptic terms are high are required (Collingridge et al. 2010; Rolls 2016a; Rolls and Treves 1990, 1998; Treves and Rolls 1991). The long-term depression can effectively remove the effect of the positive-only firing rates in the brain by subtracting the mean firing rate. Simulations that are consistent with the analytic theory have been performed (Rolls 2012a; Rolls et al. 1997b; Rolls and Webb 2012; Simmen et al. 1996).

Several points that arise, including the measurement of the total amount of information (in bits per synapse) that can be retrieved from the network, the computational definition of a memory, the computational sense in which CA3 is an attractor network and the possible computational utility of memory reconsolidation, are treated elsewhere (Rolls 2016a). Here, I note that, given that the memory capacity of the hippocampal CA3 system is limited, some form of forgetting is needed in this store or some other mechanism to ensure that its capacity is not exceeded. Exceeding the capacity can lead to a loss of much of the information retrievable from the network. Heterosynaptic LTD could help this forgetting, by enabling new memories to overwrite old memories (Rolls 2016a). The limited capacity of the CA3 system also provides one of the arguments that some transfer of information from the hippocampus to neocortical memory stores are useful (see Treves and Rolls 1994). Given its limited capacity, the hippocampus might be a useful store (e.g., for episodic memories) for only a limited period, which might be in the order of days, weeks or months. This period may well depend on the acquisition rate of new episodic memories. If the animal were in a constant and limited environment, then as new information is not being added to the hippocampus, the representations in the hippocampus would remain stable and persistent. These hypotheses have clear experimental implications, both for recordings from single neurons and for the gradient of retrograde amnesia, both of which might be expected to depend on whether the environment is stable or frequently changing. They show that the conditions under which a gradient of retrograde amnesia might be demonstrable would be when large numbers of new memories are being acquired, not when only a few memories (a few in the case of the hippocampus being less than a few hundred) are being learned (Rolls 2016a).

#### Recall and completion

A fundamental property of the autoassociation model of the CA3 recurrent collateral network is that the recall can be symmetric, i.e., the whole of the memory can be retrieved and completed from any part (Amit 1989; Hopfield 1982; Kesner and Rolls 2015; Rolls 2016a; Rolls and Treves 1998). For example, in an object-place autoassociation memory, a place can be recalled from an object retrieval cue (and potentially vice versa). In a test of this, Day et al. (2003) trained rats in a study phase to learn, in one trial, an association between two flavours of food and two spatial locations. During a recall test phase, they were presented with a flavor that served as a cue for the selection of the correct location. They found that injections of an NMDA receptor blocker (AP5) or AMPA/kainate receptor blocker (CNQX) to the dorsal hippocampus prior to the study phase impaired encoding but that injections of AP5 prior to the test phase did not impair the place recall, whereas injections of CNQX did impair the place recall. The interpretation is that, somewhere in the hippocampus, NMDA receptors are necessary for forming one-trial odour-place associations and that recall can be performed without further involvement of NMDA receptors. The implication, consistent with investigations of LTP (Takeuchi et al. 2014), is that NMDA glutamate receptors are necessary for synaptic modification but that recall may use the AMPA receptors modified by the learning.

Evidence that the CA3 system is not necessarily required during recall in a reference memory (previously learned) spatial task, such as the water maze spatial navigation for a single spatial location task, is that CA3 lesioned rats are not impaired during recall of a previously learned water maze task (Brun et al. 2002; Florian and Roullet 2004). However, if completion from an incomplete cue is needed (e.g., finding a place with only a few room cues), then CA3 NMDA receptors are necessary (presumably to ensure satisfactory CA3-CA3 learning) even in a reference memory task (Gold and Kesner 2005; Kesner and Rolls 2015; Nakazawa et al. 2002). Thus, the CA3 system appears to be especially needed in rapid one-trial object-place recall and when completion from an incomplete cue is required (see below). Note that an object-place task is a model of episodic memory, episodic memory usually has a spatial component and place cells in rats and spatial view cells in primates provide the spatial representation that is needed (Rolls 2016a; E.T. Rolls and S. Wirth in preparation).

#### Continuous spatial patterns and CA3 representations

The finding that spatial patterns, which imply continuous representations of space, are represented in the hippocampus has led to the application of continuous attractor models to help us to understand hippocampal function. This has been necessary, because (1) space is inherently continuous, (2) the firing of place and spatial view cells is approximately Gaussian as a function of the distance away from the preferred spatial location, (3) these cells have spatially overlapping fields and (4) the theory is that these cells in CA3 are connected by Hebb-modifiable synapses. This specification would inherently lead the system to operate as a continuous attractor network. Continuous attractor network models have been extensively studied (Amari 1977; Battaglia and Treves 1998a; Rolls and Stringer 2005; Samsonovich and McNaughton 1997; Stringer and Rolls 2002; Stringer et al. 2004, 2002a, b) and are described briefly next (see also Rolls 2016a).

**Fig. 5:**
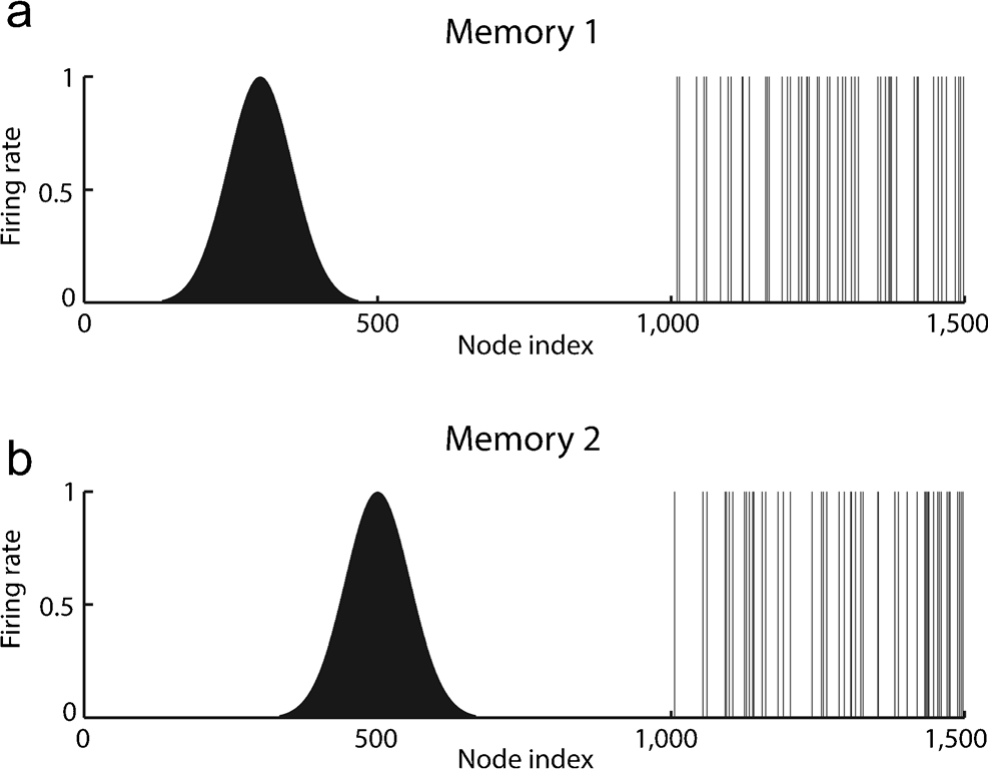
Types of firing patterns stored in continuous attractor networks illustrated for the patterns present on neurons 1–1000 for Memory 1 (when the firing is that produced when the spatial state represented is that for location 300) and for Memory 2 (when the firing is that produced when the spatial state represented is that for location 500). The continuous nature of the spatial representation results from the fact that each neuron has a Gaussian firing rate that peaks at its optimal location. This particular mixed network also contains discrete representations that consist of discrete subsets of active binary firing rate neurons in the range 1001–1500. The firing of these latter neurons can be thought of as representing the discrete events that occur at the location. Continuous attractor networks by definition contain only continuous representations, although this particular network can store mixed continuous and discrete representations and is illustrated to show the difference of the firing patterns normally stored in separate continuous attractor and discrete attractor networks. For this particular mixed network, during learning, Memory 1 is stored in the synaptic weights, then Memory 2, etc. and each memory contains a part that is continuously distributed to represent physical space and a part that represents a discrete event or object. The spatial and object representations are bound together by being simultaneously present when the event is stored (from Rolls et al. 2002, where further details can be found)

A “continuous attractor” neural network (CANN) can maintain the firing of its neurons to represent any location along a continuous physical dimension such as spatial view, spatial position and head direction. The network architecture is the same as that illustrated in Supplementary Material Box 1 for a discrete attractor network but each neuron has a peak of firing that gradually falls off the further away that the current position is from the centre of the spatial field and each neuron has a spatial field that is offset from its neighbours’ in the state space, as illustrated in Fig. 5. The CANN uses the excitatory recurrent collateral connections between the neurons as set up by associative learning to reflect the distance between the neurons in the state space of the animal (e.g., place or spatial view or head direction). These networks can maintain the bubble or packet of neural activity constant for long periods, wherever it is started to represent the current state (head direction, position, etc) of the animal and are likely to be involved in many aspects of spatial processing and memory, including spatial vision and navigation. Global inhibition is used to keep the number of neurons in a bubble or packet of actively firing neurons relatively constant and to help to ensure that only one activity packet is present (see example in Fig. 5).

Continuous attractor networks can be thought of as being very similar to autoassociation or discrete attractor networks (Rolls 2016a) and have the same architecture (Supplementary Material Box 1). The main difference is that the patterns stored in a CANN are continuous patterns, with each neuron having broadly tuned firing that decreases with, for example, a Gaussian function as the distance from the optimal firing location of the cell is varied and with different neurons having tuning that overlaps throughout the space. Such tuning is illustrated in Fig. 5. The connections set up by associative learning between the neurons in the bubble of activity can later maintain that bubble of activity (Rolls 2016a). For comparison, autoassociation networks normally have discrete (separate) patterns (each pattern implemented by the firing of a particular subset of the neurons that can overlap with other subsets), with no continuous distribution of the patterns throughout the space (see Fig. 5). A discrete sparse distributed representation with graded firing rates is used to encode and store representations of objects (Rolls 2016a; Rolls and Treves 2011). A consequent difference from a discrete attractor network is that the CANN can maintain its firing at any location in the trained continuous space, whereas a discrete attractor or autoassociation network moves its population of active neurons towards one of the previously learned separate attractor states and thus implements the recall of a particular previously learned pattern from an incomplete or noisy (distorted) version of one of the previously learned patterns.

Space is continuous and object representations are discrete. If these representations are to be combined in, for example, an object-place memory (Leutgeb et al. 2005; Rolls and Xiang 2005, 2006; Rolls et al. 2005), then we need to understand the operation of networks that combine these representations. Rolls et al. (2002) showed that attractor networks can store both continuous patterns and discrete patterns (as illustrated in Fig. 5) and can thus be used to store, for example, the location in a (continuous, physical) space (e.g., the place “out there” in a room represented by spatial view cells) where an object (a discrete item) is present. We demonstrated this by storing associated continuous and discrete representations in the same single attractor network and then by revealing that the representation in the continuous space can be retrieved by the discrete object that is associated with that spatial position and that the representation of the discrete object can be retrieved by providing the position in the continuous representation of space.

If spatial representations are stored in the hippocampus, an important issue arises in terms of understanding memories that include a spatial component or context of how many such spatial representations can be stored in a continuous attractor network. One very interesting result is that, because correlations between the representations of places in different maps or charts (where each map or chart might be of one room or locale by using, for example, cues in the room) are generally low, very many different maps can be simultaneously stored in a continuous attractor network (Battaglia and Treves 1998a; Rolls 2016a).

We considered how spatial representations can be stored in continuous attractor networks and how the activity can be maintained at any location in the state space in a form of short-term memory when the external (e.g., visual) input is removed. However, many networks with spatial representations in the brain can be updated by internal self-motion (i.e., idiothetic) cues even when no external (e.g., visual) input is present. The ways in which path integration can be implemented in recurrent networks such as the CA3 system in the hippocampus or in related systems including the entorhinal cortex (see below) are described elsewhere (Giocomo et al. 2011; McNaughton et al. 2006; Samsonovich and McNaughton 1997; Stringer et al. 2002a, 2002b) and have been applied to primate spatial view cells by Rolls and colleagues (Rolls and Stringer 2005; Stringer et al. 2004, 2005). “Cognitive maps” (O’Keefe and Nadel 1978) can be understood by the operation of these attractor networks and the way that they are updated by learning and by self-motion (Rolls 2016a). However, those who have focused on spatial and navigation processing in the hippocampus rather than memory processing do now envisage that attractor networks are involved in hippocampal function (Hartley et al. 2014).

#### Mossy fibre inputs to the CA3 cells

We hypothesize that the mossy fibre inputs force efficient information storage by virtue of their strong and sparse influence on the CA3 cell firing rates, in order to produce pattern separation, as described next. The strong effects likely to be mediated by the mossy fibres have also been emphasized by McNaughton and Morris (1987) and McNaughton and Nadel (1990). We (Rolls 1987, 1989c, 2013a, 2016a; Rolls and Treves 1998; Treves and Rolls 1992) hypothesize that the mossy fibre input is particularly appropriate in several ways. First, the finding that mossy fibre synapses are large and located very close to the soma makes them relatively powerful in activating the postsynaptic cell. Second, the firing activity of dentate granule cells appears to be very sparse (Jung and McNaughton 1993; Leutgeb et al. 2007; Neunuebel and Knierim 2012) and this, together with the small number of connections on each CA3 cell, produces a sparse signal that can then be transformed into sparse firing activity in CA3 by a threshold effect. The hypothesis is that the mossy fibre sparse connectivity solution performs the appropriate function to enable learning to operate correctly in the CA3 to CA3 synaptic connections (Cerasti and Treves 2010; Treves and Rolls 1992). Quantitative analysis shows that the perforant path input would not produce a pattern of firing in CA3 that contains sufficient information for learning (Treves and Rolls 1992).

The particular property of the small number of mossy fibre connections onto a CA3 cell, approximately 46 (see Fig. 4), is that this has a randomizing effect on the representations set up in CA3, so that they are as different as possible from each other (Cerasti and Treves 2010; Rolls 1989b, 2013a, 2016a; Rolls and Treves 1998; Treves and Rolls 1992). This is a pattern separation effect, which means, for example, that place cells in a given environment are well separated to cover the whole space and that any new object-place associations formed are different from earlier episodic memories. The result is that any one event or episode will set up a representation that is very different from other events or episodes, because the set of CA3 neurons activated for each event is random. This is then the optimal situation for the CA3 recurrent collateral effect to operate, because it can then associate together the random set of neurons that are active for a particular event (for example, an object in a particular place) and later recall the whole set from any part. It is because the representations in CA3 are unstructured or random, in this way, that large numbers of memories can be stored in the CA3 autoassociation system and that interference between the different memories is kept as low as possible, in that these memories are maximally different from each other (Hopfield 1982; Rolls and Treves 1998; Treves and Rolls 1991). If some stored memory patterns were similar, they would tend to interfere with each other during recall. For an episodic memory, each stored memory pattern should be different from the others, so that each episode can be separately retrieved.

The requirement for a small number of mossy fibre connections onto each CA3 neuron applies not only to discrete (Treves and Rolls 1992) but also to spatial representations and some learning in these connections, whether associative or not, can help to select out the small number of mossy fibres that may be active at any one time in order to chose a set of random neurons in the CA3 (Cerasti and Treves 2010). Any learning may help by reducing the accuracy required for a particular number of mossy fibre connections to be specified genetically onto each CA3 neuron. The optimal number of mossy fibres for the best information transfer from dentate granule cells to CA3 cells is in the order of 35–50 (Cerasti and Treves 2010; Treves and Rolls 1992). The mossy fibres also make connections useful for feedforward inhibition (Acsady et al. 1998), which may help to normalize the inputs and to help stability (Rolls 2016a).

On the basis of these and other points, we predicted that the mossy fibres may be necessary for new learning in the hippocampus but may not be necessary for the recall of existing memories from the hippocampus; existing memories can instead be implemented by the perforant path synapses that come directly from the entorhinal cortex and that make many more connections onto each CA3 neuron and are associatively modifiable (Rolls 2016a; Rolls and Treves 1998; Treves and Rolls 1992; see below). Experimental evidence consistent with this prediction about the role of the mossy fibres in learning has been found in rats with disruption of the dentate granule cells (Lassalle et al. 2000; see Tests of the theory).

We (Rolls and Kesner 2006) hypothesized that the nonassociative plasticity of mossy fibres (i.e., synaptic potentiation that does not depend on the activity of the postsynaptic neuron; see Brown et al. 1990) might have a useful effect in enhancing the signal-to-noise ratio of the effects of the dentate input to CA3 in that a consistently firing mossy fibre would produce nonlinearly amplified currents in the postsynaptic cell, an effect that would not happen with an occasionally firing fibre (Treves and Rolls 1992). This plasticity and the competitive learning in the dentate granule cells would also have the effect that similar fragments of each episode (e.g., the same environmental location) recurring on subsequent occasions would be more likely to activate the same population of CA3 cells. This would have potential advantages in terms of economy of use of the CA3 cells in different memories and in making some link between different episodic memories with a common feature, such as the same location in space. Consistent with this, dentate neurons that fire repeatedly are more effective in activating CA3 neurons (Henze et al. 2002).

As acetylcholine turns down the efficacy of the recurrent collateral synapses between CA3 neurons (Giocomo and Hasselmo 2007; Hasselmo and Sarter 2011; Hasselmo et al. 1995; Newman et al. 2012), then cholinergic activation also might help to allow external inputs rather than the internal recurrent collateral inputs to dominate the firing of the CA3 neurons during learning, as the current theory proposes (Rolls 2013a; Rolls and Deco 2015). If cholinergic activation at the same time facilitated LTP in the recurrent collaterals (as it appears to in the neocortex), then cholinergic activation might have a useful double role in facilitating new learning at times of behavioural activation and emotional arousal, when presumably it may be particularly relevant to allocate some of the limited memory capacity to new memories. Acetylcholine may also facilitate memory storage (versus recall) by enhancing firing in dentate granule cells (see Kesner and Rolls (2015)).

#### Perforant path inputs to CA3 cells

By calculating the amount of information that would end up being carried by a CA3 firing pattern produced solely by the perforant path input and by the effect of the recurrent connections (i.e., without dentate input), we showed (Treves and Rolls 1992) that an input of the perforant path type, alone, is unable to direct efficient information storage. Such an input is too weak, it turns out, to drive the firing of the cells, as the “dynamics” of the network is dominated by the randomizing effect of the recurrent collaterals. On the other hand, an autoassociative memory network needs afferent inputs to apply the retrieval cue to the network. We have shown that the perforant path system is likely to be the one involved in relaying the cues that initiate retrieval in CA3. The concept is that, in order to initiate retrieval, a numerically large input through associatively modified synapses is useful, so that even a partial cue is sufficient and that the retrieval cue need not be very strong, as the recurrent collaterals then take over in the retrieval process (Rolls 2016a; Treves and Rolls 1992). In contrast, during storage, strong signals, in the order of millivolts for each synaptic connection, are provided by the mossy fibre inputs to dominate the recurrent collateral activations, so that the new pattern of CA3 cell firing can be stored in the CA3 recurrent collateral connections (Rolls 2016a; Treves and Rolls 1992).

The associatively modified synapses required in the perforant path to CA3 synapses make this a pattern association network. The architecture and properties of pattern association networks are described briefly in Supplementary Material Box 2 and in more depth elsewhere (Rolls 2016a). These synapses need to be modified during the storage of an event memory, with the entorhinal input to CA3 becoming associated with whatever subset of neurons in CA3 is firing at that time (Treves and Rolls 1992).

#### Noise in memory recall

Randomness (sometimes referred to as noise) is present in the spiking times of individual neurons, i.e., for a given mean firing rate, the spike times often have a close to Poisson distribution. The noise arises from synaptic and neuronal processes in ion channels, the quantal release of transmitter, etc. (Faisal et al. 2008). A result is that, in an autoassociation network, if one population of neurons for one attractor or memory state has by chance more spikes from its neurons than the other populations, then the memory with more spikes is more likely to be recalled, especially when the recall cue or cues for the various neurons are relatively similar in strength. The operation of such systems has been described in *The Noisy Brain: Stochastic Dynamics as a Principle of Brain Science* (Rolls and Deco 2010) in the context of decision-making (Wang 2008) but the approach applies equally to memory recall in an autoassociation memory, as the network architecture and operation for memory and for decision-making is the same (Rolls 2016a). This noisy operation of the brain has been proposed to have many advantages, for example, in promoting the recall of different memories or different associations on different occasions, even when the inputs are similar and this is proposed to be an important contributor to original thought and creativity (Rolls 2016a; Rolls and Deco 2010). Too much noise and therefore the instability of memory and decision systems might promote unstable attention and loose thought associations in schizophrenia (Loh et al. 2007; Rolls 2012b; Rolls et al. 2008b). Too little noise and therefore too much stability may contribute to some of the symptoms of obsessive-compulsive disorder (Rolls 2012b; Rolls et al. 2008a). In both these cases, the combination of theoretical neuroscience approaches with experimental evidence concerning the transmitters present in these states is leading to interesting new approaches to understanding these disorders and perhaps to treating them more successfully (Rolls 2012b, 2016a), together with the cognitive effects in normal aging (Rolls and Deco 2015).

### Dentate granule cells

#### Pattern separation

We now turn to the hypothesis that the dentate granule cell stage of hippocampal processing, which precedes the CA3 stage, acts as a competitive network in a number of ways to produce, during learning, the sparse yet efficient (i.e., non-redundant) representation in CA3 neurons that is required for the autoassociation implemented by CA3 to perform well (Rolls 1989b, 2016a; Rolls et al. 2006; Treves and Rolls 1992). The properties of competitive networks are summarized in Supplementary Material Box 3 and in more detail by Rolls (2008, 2016a). An important property for episodic memory is that the dentate, by acting in this way, performs pattern separation (or orthogonalization; Rolls 1989b, 2013a; Rolls et al. 2006; Treves and Rolls 1992), enabling the hippocampus to store different memories of even similar events; this prediction has been confirmed (Gilbert et al. 2001; Goodrich-Hunsaker et al. 2008; Kesner and Rolls 2015; Leutgeb and Leutgeb 2007; McHugh et al. 2007; Rolls 2016a; see also Tests of the theory). The term pattern separation refers to the property that the output patterns are less correlated with each other than the input patterns, i.e., orthogonaliztion has been produced.

As just described, the dentate granule cells might be important in helping to build and prepare spatial representations for the CA3 network. The actual representation of space in the primate hippocampus includes a representation of spatial view (E.T. Rolls and S. Wirth in preparation; Rolls and Xiang 2006), whereas in the rat hippocampus, it is of the place where the rat is. The representation in the rat may be related to the fact that, with a much less developed visual system than the primate, the rat’s representation of space may be defined more by the olfactory, tactile and distant visual cues present and may thus tend to reflect the place in which the rat finds itself. However, the spatial representations in the rat and primate could arise from essentially the same computational process as follows (de Araujo et al. 2001; Rolls 1999). The starting assumption is that, in both the rat and the primate, the dentate granule cells (and the CA3 and CA1 pyramidal cells) respond to combinations of the inputs received. In the case of the primate, a combination of visual features in the environment will result, because of the fovea providing high spatial resolution over a typical viewing angle of perhaps 10–20 degrees, in the formation of a spatial view cell, the effective trigger for which will thus be a combination of visual features within a relatively small part of space. In contrast, in the rat, given the very extensive visual field that is subtended by the rodent retina and that may extend over 180–270 degrees, a combination of visual features formed over such a wide visual angle would effectively define a position in space that is a place (de Araujo et al. 2001).

#### Entorhinal cortex grid cells transformed to hippocampal place and spatial view cells

**Fig. 6:**
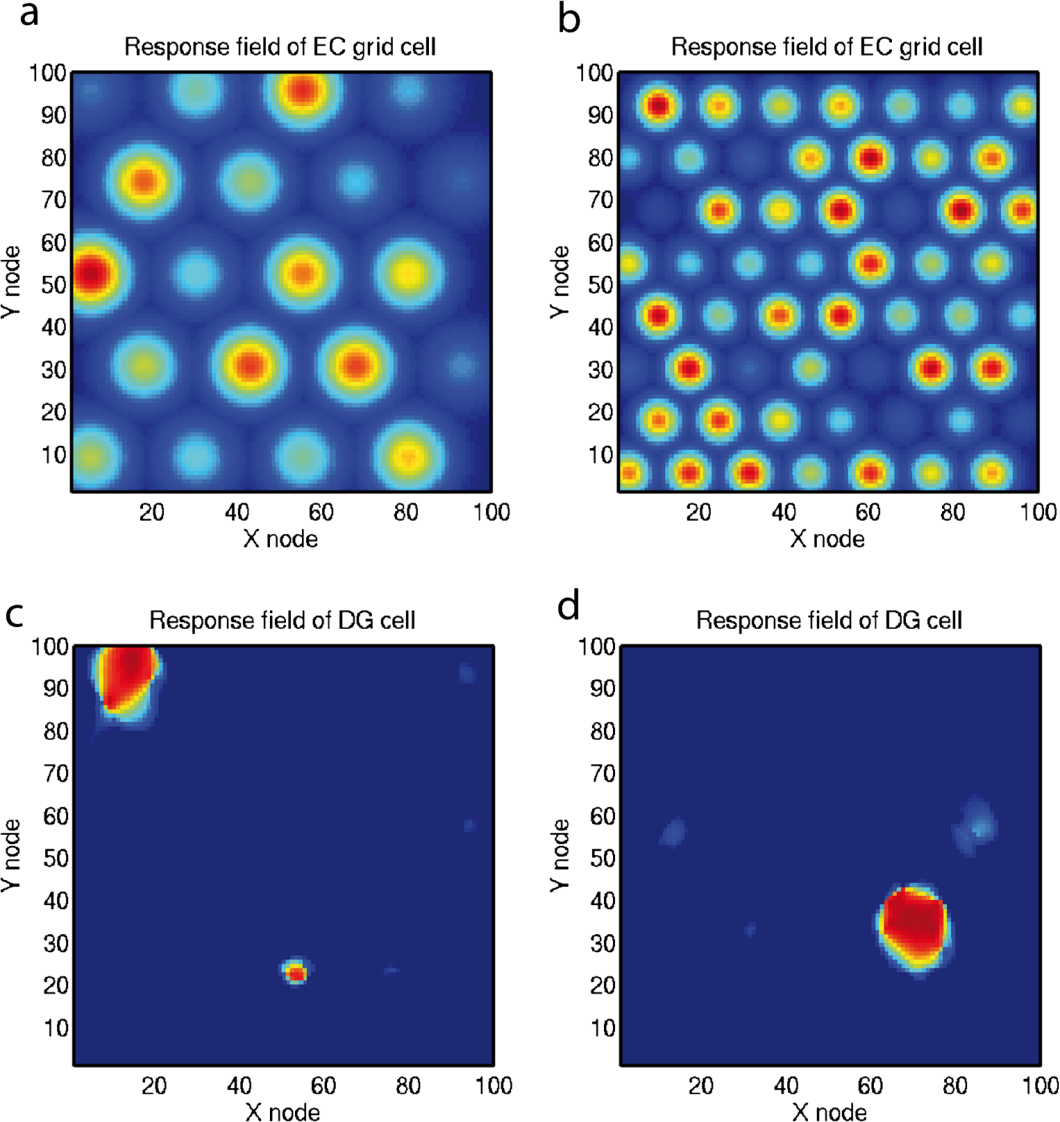
Simulation of competitive learning in the dentate gyrus to produce place cells from the entorhinal cortex grid cell inputs. **a**, **b** Firing rate profiles of two entorhinal cortex (*EC*) grid cells with frequencies of 4 and 7 cycles. The colours show the firing rates with *blue* being the lowest and *red* the highest in the test environment (e.g., a room) in which the spatial coordinates are *X* and *Y*. **c**, **d** Firing rate profiles of two dentate gyrus (*DG*) cells after competitive learning. After Rolls et al. (2006)

The entorhinal cortex contains grid cells that have a high firing rate in the rat in a two-dimensional (2D) spatial grid as the rat traverses an environment, with larger grid spacings in the ventral entorhinal cortex (Moser et al. 2015). This may be a system optimized for path integration (McNaughton et al. 2006), which may self-organize during locomotion with longer time constants producing more widely spaced grids in the ventral entorhinal cortex (Kropff and Treves 2008). How are the grid cell representations, which would not be suitable for the association of an object or reward with a place to form an episodic memory, transformed into a place representation that would be appropriate for this type of episodic memory? I have proposed that this might be implemented by a competitive network (Rolls 2016a) in the dentate gyrus operating to form place cells and implemented by each dentate granule cell learning to respond to particular combinations of entorhinal cortex cells firing, where each combination effectively specifies a place; this has been shown to be feasible computationally (Rolls et al. 2006). The sparse representations in the dentate gyrus, implemented by the mutual inhibition through inhibitory interneurons and competitive learning, help to implement this “pattern separation” effect (Rolls 1989b, 1989c, 2016a; Rolls and Treves 1998). Results of this competitive learning model are illustrated in Fig. 6. Figure 6a, b show simulated (entorhinal cortex) grid cells with Gaussian firing rate response profiles, whereas Fig. 6c, d illustrate the place cells formed in the hippocampus by competitive learning (Rolls et al. 2006). Similar processes are involved in some later models of this transformation (Giocomo et al. 2011; Zilli 2012).

In primates, there is now evidence for the presence of a grid-cell like representation in the entorhinal cortex, with neurons having grid-like firing as the monkey moves its eyes across a spatial scene (Buffalo 2015; Killian et al. 2012; Rueckemann and Buffalo 2017). Similar competitive learning processes may transform these “spatial view grid cells” of the entorhinal cortex into hippocampal spatial view cells and may help with the idiothetic (produced in this case by movements of the eyes) update of spatial view cells (Robertson et al. 1998).

Spatial view cells in primates represent a scene view allocentrically, as described below. How could such spatial view representations be formed in which the relative spatial position of features in a scene is encoded? I have proposed that this involves competitive learning analogous to that used to form place cells in rats but, in primates, operating on the representations of objects that reach the hippocampus from the inferior temporal visual cortex (Rolls et al. 2008c). We have shown that, in complex natural scenes, the receptive fields of inferior temporal cortex neurons become reduced in size and asymmetric with respect to the fovea (Aggelopoulos and Rolls 2005; Rolls 2009) and we have demonstrated, in a unifying computational approach, that competitive network processes operating in areas such as the parahippocampal cortex, the entorhinal cortex and/or the dentate granule cells might form unique views of scenes by forming a sparse representation of these object or feature-tuned inferior temporal cortex ventral visual stream representations that have some spatial asymmetry providing a foundation for building scene representations that incorporate the relative spatial positions of landmarks within a scene (Rolls et al. 2008c). In this theory, it is the spatial asymmetry with respect to the fovea of different neurons that solves the binding problem, for the neurons indeed respond to an object and to its location with respect to the fovea (Aggelopoulos and Rolls 2005; Rolls 2009; Rolls et al. 2008c). Another input to hippocampal spatial view cells may come from the parahippocampal place area (Nasr et al. 2011).

### CA1 cells

The CA3 cells connect to the CA1 cells by the Schaeffer collateral synapses. The associative modifiability in this connection helps the full information present in CA3 to be retrieved in the CA1 neurons (Rolls 1995; Schultz and Rolls 1999; Treves 1995; Treves and Rolls 1994). Part of the hypothesis is that the various sub-parts of an episodic memory, which have to be represented separately in CA3 to allow for completion, can be combined together by competitive learning in CA1 to produce an efficient retrieval representation for the recall via the backprojection pathways to the neocortex of memories stored in the neocortex (Rolls 1989b, 2016a; Treves and Rolls 1994).

### Backprojections to the neocortex and memory recall

The need for information to be retrieved from the hippocampus to affect other brain areas was noted in the Introduction. The way in which this could be implemented via backprojections to the neocortex is now considered.

The modifiable connections from the CA3 neurons to the CA1 neurons have been suggested to allow the whole episode in CA3 to be produced in CA1. The CA1 neurons would then activate, via their termination in the deep layers of the entorhinal cortex, at least the pyramidal cells in the deep layers of the entorhinal cortex (see Fig. 2). These entorhinal cortex layer 5 neurons would then, by virtue of their backprojections (Lavenex and Amaral 2000; Witter et al. 2000) to the parts of the cerebral cortex that originally provided the inputs to the hippocampus, terminate in the superficial layers (including layer 1) of those neocortical areas, where synapses would be made onto the distal parts of the dendrites of the (superficial and deep) cortical pyramidal cells (Rolls 1989a, 1989b, 1989c, 2016a; see Fig. 2). The areas of neocortex in which this recall would be produced could include multimodal cortical areas (e.g., the cortex in the superior temporal sulcus, which receives inputs from temporal, parietal and occipital cortical areas and from which it is thought that cortical areas such as 39 and 40 related to language have developed) and also areas of unimodal association cortex (e.g., inferior temporal visual cortex). The backprojections, by recalling previous episodic events, could provide information useful to the neocortex in the building of new representations in the multimodal and unimodal association cortical areas, which by building new long-term and structured representations can be considered as a form of memory consolidation (Rolls 1989a, 1989b, 1989c, 1990a, b, 2016a), or in organizing actions.

The hypothesis of the architecture whereby this multistage recall from the hippocampus to the neocortex is achieved is shown in Fig. 2. The feedforward connections from association areas of the cerebral neocortex (solid lines in Fig. 2) show major convergence as information is passed to CA3, with the CA3 autoassociation network having the smallest number of neurons at any stage of the processing. The backprojections allow for divergence back to neocortical areas. The way in which I suggest that the backprojection synapses are set up to have the appropriate strengths for recall is as follows (Kesner and Rolls 2015; Rolls 1989a, 1989b, 1989c, 2016a). During the setting up of a new episodic memory, strong feedforward activity progresses towards the hippocampus. During the episode, the CA3 synapses are modified and, via the CA1 neurons and the subiculum, a pattern of activity is produced on the backprojecting synapses to the entorhinal cortex. Here, the backprojecting synapses from active backprojection axons onto pyramidal cells, being activated by the forward inputs to entorhinal cortex, are associatively modified. A similar process would be implemented at preceding stages of the neocortex, i.e., in the parahippocampal gyrus/perirhinal cortex stage and, in association, cortical areas, as shown in Fig. 2.

The concept is that, during the learning of an episodic memory, cortical pyramidal cells in at least one of the stages would be driven by forward inputs from earlier cortical areas but would simultaneously receive backprojected activity (indirectly) from the hippocampus. This activity would, by pattern association from the backprojecting synapses to the cortical pyramidal cells, become associated with whichever cortical cells were being made to fire by the forward inputs. Then, later on, during recall, a recall cue from perhaps another part of the neocortex might reach CA3, where the firing during the original episode would be completed. The resulting backprojecting activity would then, as a result of the pattern association learned previously in the hippocampo-cortical backprojections, bring back the firing in any cortical area that was present during the original episode. Thus, retrieval involves the reinstating of the neuronal activity that was present in different cortical areas and that was present during the learning of an episode. The pattern association is also called heteroassociation in order to contrast it with autoassociation. The pattern association operates at multiple stages in the backprojection pathway, as is made evident in Fig. 2. If the recall cue was an object, this might result in the recall of the neocortical firing that represented the place in which that object had been seen previously. As noted elsewhere in this review and by McClelland et al. (1995), that recall might be useful to the neocortex to help it build new semantic memories, which might inherently be a slow process and is not part of the theory of recall from the hippocampus (see below).

Overall, this is thus a theory of the way that different events, linked together in CA3 during the formation of an episodic memory, could produce completion in CA3 if only one of those events is presented later in recall. This would then in turn via CA1 address, by multistage pattern association, the cortical areas in which activity was present during the original learning of the episodic memory and would reinstate the neocortical neuronal activity that was present when the episodic memory was formed. This theory is supported by a computational neuroscience model of the operation of the whole of this system (Rolls 1995).

A plausible requirement for a successful hippocampo-directed recall operation is that the signal generated from the hippocampally retrieved pattern of activity and carried backwards towards the neocortex remains undegraded when compared with the noise attributable, at each stage, to the interference effects caused by the concurrent storage of other patterns of activity on the same backprojecting synaptic systems. This requirement is equivalent to that used in deriving the storage capacity of such a series of heteroassociative memories; Treves and Rolls (1991, 1994) showed that the maximum number of independently generated activity patterns that can be retrieved is given, essentially, by the same formula as above where, however, *a* is now the sparseness of the representation at any given stage and *C* is the average number of (back-)projections that each cell of that stage receives from cells of the previous one (k’ is a similar slowly varying factor to that introduced above). If *p* is equal to the number of memories held in the hippocampal memory, it is limited by the retrieval capacity of the CA3 network, *p*_max_. Putting together the formula for the latter with that shown here, one concludes that, roughly, the requirement implies that the number of afferents of (indirect) hippocampal origin to a given neocortical stage (*C* ^HBP^), must be *C* ^HBP^ = *C* ^RC^ *a*_nc_/*a*_CA3_, where *C* ^RC^ is the number of recurrent collaterals to any given cell in CA3, the average sparseness of a representation is *a*_nc_ and *a*_CA3_ is the sparseness of memory representations in CA3 (HBP refers to hippocampal back projections; the implication of the argument is that there must be very many backprojection synapses onto each neocortical neuron; Treves and Rolls 1994).

The above requirement is very strong: even if representations were to remain as sparse as they are in CA3, which is unlikely, to avoid degrading the signal, *C* ^HBP^ should be as large as *C* ^RC^, i.e., 12,000 in the rat. If then *C* ^HBP^ has to be of the same order as *C* ^RC^, one is led to a very definite conclusion: a mechanism of the type envisaged here could not possibly rely on a set of monosynaptic CA3-to-neocortex backprojections. This would imply that, to make a sufficient number of synapses on each of the vast number of neocortical cells, each cell in CA3 has to generate a disproportionate number of synapses (i.e., *C* ^HBP^ times the ratio between the number of neocortical and that of CA3 cells). The required divergence can be kept within reasonable limits only by assuming that the backprojecting system is polysynaptic (i.e., involves several connected cortical stages), provided that the number of cells involved grows gradually at each stage, from CA3 back to neocortical association areas (Treves and Rolls 1994; cf. Fig. 2).

The theory of recall by the backprojections thus provides a quantitative account of why any neocortical area has as many backprojection as forward projection connections. Further aspects of the operation of the backprojecting systems are described elsewhere (Rolls 2016a).

The theory described by McClelland et al. (1995) is similar to the theory described above, except that it holds that the last set of synapses that are modified rapidly during the learning of each episode are those between the CA3 and the CA1 pyramidal cells (see Fig. 2). Their theory also emphasizes the important point that the hippocampal and neocortical memory systems may be quite different, with the hippocampus being specialized for the rapid (“one-shot”) learning of unstructured single events or episodes and the neocortex for the slower learning of semantic representations (structured representations in which the components are linked), which may necessarily benefit from the many exemplars needed to shape the semantic representation, a process that is helped by the recall of episodic memories from the hippocampus. The particular model on which they focus for the learning of semantic representations by interleaved learning is the connectionist model of Rumelhart (Rumelhart 1990; Rumelhart and Todd 1993), which is trained by error backpropagation (Rumelhart et al. 1986).

### Temporal order memory in the hippocampus and episodic memory

For some time, evidence has been available that the hippocampus plays a role in temporal order memory, perhaps for a sequence of spatial locations but also even when there is no spatial component (Kesner and Rolls 2015). In humans, the hippocampus becomes activated when the temporal order of events is being processed (Lehn et al. 2009) and temporal context is important in episodic memory (Howard et al. 2012). One approach regarding the way that the hippocampus might be involved in temporal order memory is by encoding temporal order into each gamma cycle nested into a theta cycle (Lisman and Buzsaki 2008; Lisman and Redish 2009). A very different approach is to use firing rate encoding in attractor networks (Rolls 2010; Rolls and Deco 2010) and is based on evidence that neurons in the rat hippocampus have firing rates that reflect which temporal part of the task is current (Macdonald et al. 2011). In particular, a sequence of different neurons is activated at successive times during a time delay period. The tasks used include an object-odour paired associate non-spatial task with a 10 s delay period between the visual stimulus and the odour. The evidence also shows that a large proportion of hippocampal neurons fire in relation to individual events in a sequence being remembered (e.g., a visual object or odour) and some to combinations of the event and the time in the delay period (Eichenbaum 2014; Macdonald et al. 2011).

These interesting neurophysiological findings indicate that rate encoding is being used to encode time, i.e., the firing rates of different neurons are high at different times within a trial, delay period, etc. (Eichenbaum 2014; Macdonald et al. 2011). These findings suggest several possible computational processes (Kesner and Rolls 2015; Rolls 2010).

First, because some neurons fire at different times in a trial of a temporal order memory task or delay task, the time in a trial at which an object (e.g., a visual stimulus or odour) was presented could become encoded in the hippocampus by an association implemented in the CA3 recurrent collaterals between the neurons that represent the object (previously known to be present in the hippocampus for tasks for which the hippocampus is required; Rolls and Xiang 2006; Rolls et al. 2005) and the “time encoding” neurons in the hippocampus (Macdonald et al. 2011). This would allow associations for the time at which the object was present to be formed. Given that time encoding neurons are also found in the medial entorhinal cortex (Kraus et al. 2013a), this could provide the source of the time information required by CA3. However, although lesions of CA3 impair temporal order-place representations, it is lesions of CA1 that impair temporal order-visual object and temporal order-odour representations (Kesner and Rolls 2015). Thus, temporal timing and object information is possibly brought together by competitive learning in CA1 (Kesner and Rolls 2015), which receives inputs not only from CA3 but also directly from the entorhinal cortex (see Fig. 2).

Second, these associations would provide the basis for the recall of the object from the time in a trial or vice versa. The retrieval of object or temporal information from each other would occur in CA3 in a way that is analogous to that shown for recalling the object from the place or, vice versa, the place from the object (Rolls et al. 2002) but by substituting the details of the properties of the “time encoding” neurons (Eichenbaum 2014; Macdonald et al. 2011) for what was previously the spatial (place) component. Alternatively, if competitive learning in CA1 is the mechanism, generalization in the competitive learning (Rolls 2016a) from either the object or the temporal order cue would retrieve the whole representation. In addition, if the time encoding neurons simply cycled through their normal sequence during recall, this would enable the sequence of objects or events associated with each subset of time encoding neurons to be recalled correctly in the order in which they were presented.

Third, we need a theory with respect to the origin of the temporal effect, whereby different hippocampal (or potentially prefrontal cortex) neurons fire in different parts of a trial or delay period. We can consider three hypotheses about the way that the firing of the ‘time encoding’ hippocampal neurons is produced. All utilize slow transitions between attractor states that can be a property of noisy attractor networks.

The first hypothesis is that an attractor network with realistic dynamics (modelled at the integrate-and-fire level with a dynamical implementation of the neuronal membrane and synaptic current dynamics and with synaptic or neuronal adaptation) can implement a sequence memory, as shown by Deco and Rolls (2005). The hypothesis is that there are several different attractors and that weak connections exist between the different attractors. In the model, adaptation produces effects whereby, whatever sequence (order of stimuli) is presented in an individual trial, that order can be replayed in the same sequence, because as one attractor state dies as a result of the adaptation, the next attractor to emerge from the spontaneous firing because of the spiking-related noise is the one that has been active least recently and is the one that is least adapted (Deco and Rolls 2005). The whole system operates at a rather slow timescale for the transitions between the attractors, partly because of the time for the noise to drive the system from one attractor state to another and partly because of the slow time course of the adaptation (Deco and Rolls 2005; Rolls and Deco 2010). This implements a type of order memory.

The second hypothesis is analogous and is also implemented in a recurrently connected system such as the hippocampal CA3 system or local recurrent circuits in the neocortex (Rolls and Deco 2010). This second theory is that, again, there are several attractors but that each attractor is connected by slightly stronger forward than reverse synaptic weights to the next. In previous work, we have shown that, with an integrate-and-fire implementation with spiking noise, this allows slow transitions from one attractor state to the next (Deco et al. 2005; Deco and Rolls 2003). During learning of the synaptic weights in the network, adaptation might lead to each “time encoding” population of neurons responding for only a limited period, helping to produce multiple sequentially activated populations of time encoding neurons (Rolls and Deco 2010). In this scenario, stronger forward than reverse weights between different attractors each consisting of a different population of “time encoding” neurons would be the essence.

The third hypothesis is that the mechanism for the time encoding neurons lies in the entorhinal cortex where there are ring attractors, as described below.

The possibility that the recurrent collateral connections in, for example, CA3 could be used to store long sequences by employing discrete timesteps (Cheng 2013) seems implausible, for an important property of attractor networks is that when implemented with integrate-and-fire neurons, the dynamics become continuous and the whole attractor network settles very fast into its basin of attraction, in 1.5 times the constants of the synapses, i.e., within typically 20 ms, without going through discrete states (Battaglia and Treves 1998b; Panzeri et al. 2001; Rolls 2016a; Rolls and Webb 2012; Treves et al. 1997).

Temporal order memory has been suggested to be implemented in the hippocampus as described above and might make an important contribution to episodic memory in which several events linked in the correct order might form an episode. The theory shows how items in a particular temporal order could be separated from each other, a property that we have referred to as the temporal pattern separation effect (Kesner and Rolls 2015). The theory of episodic memory described here indicates ways in which events and sequences of events could be recalled from the hippocampus to the neocortex in which a longer-term more semantic representation of a recalled episode, such as what happened on one’s fifth birthday, might be stored and then accessed to describe the episode. For the order to be correctly implemented in the semantic neocortical store, a similar mechanism involving, for example, stronger forward than reverse synaptic weights between long-term memory representations in attractors might build an appropriate long-term order memory (Rolls and Deco 2010).

### Entorhinal cortex grid cells

The entorhinal cortex contains grid cells that have a high firing rate in the rat in a 2D spatial grid as the rat traverses an environment (see Fig. 6), with larger grid spacings in the ventral entorhinal cortex (Fyhn et al. 2004; Hafting et al. 2005; Moser et al. 2015). Computational approaches to this system model it as a set of linked ring continuous attractors (Giocomo et al. 2011; Kropff and Treves 2008). These are the CANNs described above. The concept is that, as the rat locomotes, the peak of the firing in the continuous attractor moves and, after a certain distance has been navigated, the place represented returns to the same set of neurons, completing the ring. The position of the peak in the ring continuous attractor is updated, for example, by self-motion or possibly by time for at least some neurons. By having different ring attractors that cover large to small distances with one pass through the ring, the system provides, with its multiscale representation, information that, when read out, appropriately provides a coarse and fine representation of position. The phases of the different ring attractors must be locked for this to work. The use of ring attractors could, in this way, implement a representation of the position of the rat in a 2D environment; this representation would be self-generating and so would work in any environment, if it is updated by self-motion or time. Indeed, one theory of the underlying mechanism is that neuronal or synaptic adaptation could be used to make the continuous attractor move its peak of activity continuously round the ring as a function of time (Kropff and Treves 2008). A fast adaptation mechanism would produce small rings for the grid, whereas a slow adaptation mechanism would produce large rings for the grid. Part of the interest in this suggestion is that grid cells formed by using this adaptation process would effectively be time cells, different cells of which would fire at different times in a trial, as have now been described in the rat entorhinal cortex (Kraus et al. 2013a) and also in the hippocampus itself (Kraus et al. 2013b, 2015). A set of various modelling approaches for the grid cells have been described by Giocomo et al. (2011). The system may be used therefore not only for spatial path integration (McNaughton et al. 2006) but also for the timing information useful in sequence encoding for non-spatial and spatial information (Kesner and Rolls 2015).

## Navigation and the hippocampus

A fundamental question about the function of the hippocampus in rodents and primates including humans is whether the hippocampus is for memory or navigation. Strong emphasis is placed on navigation as a function of rodent place cells (Burgess et al. 2000; Burgess and O’Keefe 1996; Hartley et al. 2014 O’Keefe 1979, 1991). In one approach to the function of the hippocampal system in rodents, attractor dynamics for path integration have been suggested to be implemented in the entorhinal cortex (for which the evidence is good; Giocomo et al. 2011; Kropff and Treves 2008; Moser et al. 2014), although the connectivity within the hippocampus is “preconfigured”, with the spatial inputs mapping onto this hardwired structure, which is described as a continuous spatial map (Colgin et al. 2010). External inputs are then held to learn to link correctly onto the appropriate part of this preconfigured map (Colgin et al. 2010). According to this spatial map theory of the rat hippocampus, there would be no episodic learning of associations between objects and places in hippocampal networks such as CA3 for episodic or event memory and no attractor dynamics within the hippocampus. The discovery of hippocampal cells that respond first to one location and then to another in an ambiguous visual environment is usually however taken as evidence that attractor dynamics exist within the hippocampus (Jezek et al. 2011). The purely spatial navigation approach to hippocampal function is also inconsistent with the presence of object-related information in the hippocampus, with object-place association information in the primate hippocampus, with the evidence in rats indicating that one-trial object-place associations are hippocampus-dependent (Day et al. 2003; Kesner and Rolls 2015) and with the evidence from humans implicating the hippocampus in episodic memory (Maguire et al. 2016; Zeidman and Maguire 2016).

Spatial information is almost always part of an episodic memory and thus spatial representations in the hippocampus may be useful for navigation. For example, episodic memories of particular journeys could help to build neocortical maps that would require many journeys to elaborate. Such maps may be found in the neocortex, given the evidence that lesions to the neocortex can produce topographical agnosia and the inability to navigate (Kolb and Whishaw 2015). Further, the right hippocampus in humans is activated during mental navigation in recently learned but not highly familiar environments (Hirshhorn et al. 2012). Mental navigation in familiar environments activates cortical areas, such as the lateral temporal cortex, posterior parahippocampal cortex, lingual gyrus and precuneus (Hirshhorn et al. 2012). Given these data, a consideration of the role of the hippocampus in navigation is of interest.

First, any model of navigation based on place cells in rodents cannot provide an adequate model of the role of the primate hippocampal cortex in navigation, in view of the presence of spatial view cells in primates, which by their firing provide a basis for the representation of places other than where an individual is located, i.e., for the representations of positions in scenes at which an individual is looking, even if the scene is being remembered based, for example, on idiothetic (self-motion) update (Rolls and Xiang 2006). Spatial view cells provide a basis for the representation of scenes, landmarks in scenes and locations of objects and rewards in scenes (Rolls 2016a; Rolls and Xiang 2006). This type of representation is likely to be crucial in primates, including humans, for computations involved in navigating to new places in which the individual has not been located previously. Moreover, spatial view neurons are found not only in CA3 and CA1 but also in the parahippocampal cortex (Rolls and Xiang 2006).

Second, hippocampal place cells in rodents and spatial view cells in primates can be updated by idiothetic (self-motion) inputs, for example, by moving the eyes to a different location in a scene in the dark (Robertson et al. 1998). The basis for this is the idiothetic update of attractor networks of grid cells on the entorhinal cortex (Giocomo et al. 2011). This may be useful in updating not only location for use in episodic memory but also position for use in navigation (Burgess 2008; Burgess and O’Keefe 1996; Erdem and Hasselmo 2012). These processes may employ head direction cells found in the presubiculum of rodents and primates (Robertson et al. 1999; Taube et al. 1996; Wiener and Taube 2005) and cells that respond at the boundaries of an enclosure (Lever et al. 2009).

To summarize, the evidence described in this review indicates that the hippocampus is involved in episodic unstructured memory by utilizing a single attractor network in CA3 for one-trial object-place and related associations, that the dentate system prepares the inputs for storage by performing pattern separation and that the backprojections to the neocortex are used for memory retrieval. This system might be useful in navigation, at least in new environments where episodic information may be helpful. In addition, a system of attractor networks exists in the entorhinal cortex for path integration, which may be of value for idiothetic navigation and for idiothetic update of the place being represented in the hippocampal memory system.

## Tests of the theory

A useful theory should make predictions that can then be tested to substantiate the theory or to show ways in which it should be developed or modified. This section illustrates the important and rich interplay that occurs between theory and experiment, which is essential for understanding the manner in which the brain computes. Further developments have been described (Kesner and Rolls 2015).

### Dentate granule cells

The theory predicts that the dentate granule cell mossy fibre system of inputs to the CA3 neurons is necessary to store spatial memories but not to recall them (Rolls 2016a; Treves and Rolls 1992, 1994). Lassalle et al. (2000) obtained evidence consistent with this in rats with damage to the mossy fibre system and further evidence has been provided consistent with this idea (Daumas et al. 2009; Kesner and Rolls 2015; Lee and Kesner 2004).

The theory predicts that pattern separation is performed by the dentate granule cells. Evidence consistent with this has been found neurophysiologically in the small sparsely encoded place fields of dentate neurons (Jung and McNaughton 1993; Leutgeb and Leutgeb 2007) and their reflection in CA3 neurons (Leutgeb and Leutgeb 2007). Selective dentate lesions in rats (Gilbert and Kesner 2003; Gilbert et al. 2001; Goodrich-Hunsaker et al. 2008; Kesner and Rolls 2015; Rolls 2016a) or dentate NMDA receptor knockouts in mice (McHugh et al. 2007) have been shown to impair spatial object-place (or reward-place: remembering where to find a reward) association tasks, especially when the places are close together and require pattern separation before storage in CA3.

If adult neurogenesis in the dentate gyrus (Clelland et al. 2009; Nakashiba et al. 2012) does indeed prove to be functionally relevant, its computational role could be to facilitate pattern separation for new patterns by providing new dentate granule cells with new sets of random connections to CA3 neurons. Consistent with the dentate spatial pattern separation hypothesis (Rolls 1989b, c, 1996; Treves and Rolls 1992, 1994), in mice with impaired dentate neurogenesis, spatial learning in a delayed non-matching-to-place task in the radial arm maze is impaired for arms that are presented with little separation, whereas no deficit is observed when the arms are presented farther apart (Clelland et al. 2009). Consistently, impaired neurogenesis in the dentate also produces a deficit for small spatial separations in an associative object-in-place task (Aimone and Gage 2011; Clelland et al. 2009). Neurogenesis in this system may be useful because the role of the dentate granule cell / mossy fibre system is to produce pattern separation for the CA3 representations involved in making new episodic memories very different from previous episodic memories and not to play a role by synaptic modification of the mossy fibre synapses in the retrieval of the information stored in CA3. In other cortical systems, the synapses involved in storage and recall are the same and are associatively modified (e.g., in neocortical pattern association, autoassociation and competitive learning systems; Rolls 2016a) and neurogenesis is accordingly not present.

The theory predicts that the direct perforant path input from the entorhinal cortex to the CA3 cells (which bypasses the dentate granule cells) is involved in the recall of memory from the CA3 system. Lee and Kesner (2004) obtained evidence consistent with this in a Hebb-Williams maze recall task by showing that lesions of the perforant path impair retrieval (Lee and Kesner 2004).

### Region CA3

The theory predicts that the CA3 is especially important in object-place or reward-place tasks in which associations must be formed between any spatial location and any object (referred to as “arbitrary associations”). Much evidence has been gained from subregion analyses involving the disruption of CA3 showing that CA3 is necessary for arbitrary associations between places and objects or rewards (Gilbert and Kesner 2003; Kesner and Rolls 2015). Similar impairments have been obtained following the deletion of CA3 NMDA receptors in mice in the acquisition of an odour-context paired associate learning task (Rajji et al. 2006). If place or time is not a component, associative tasks such as odour-object association are not impaired (Kesner and Rolls 2015), underlining the fact that the hippocampus is especially involved in episodic types of associative memory that typically involve place and/or time.

The theory predicts that the CA3 is especially important in object-place or reward-place completion tasks in which associations must be completed from a part of the whole. If completion from an incomplete cue is needed, then CA3 NMDA receptors have been shown to be necessary (presumably to ensure satisfactory CA3-CA3 learning), even in a reference memory task (Gold and Kesner 2005; Nakazawa et al. 2002).

The theory predicts that the CA3 system is especially needed in rapid one-trial object-place learning and recall.Hippocampal NMDA receptors (necessary for LTP to occur) are needed for one-trial flavour-place association learning and hippocampal AMPA/kainate receptors are sufficient for the recall, although the hippocampal subregion involved has not been tested (Day et al. 2003). In subregion studies, Kesner and colleagues have shown that CA3 lesions produce chance performance on a one-trial object-place recall task (Kesner et al. 2008) and other object-spatial tasks (Kesner and Rolls 2001, 2015). For example, CA3 lesions produce chance performance on both a one-trial object-place recall and a place-object recall task (Kesner et al. 2008). This is evidence that CA3 supports arbitrary associations and episodic memory based on one-trial learning. A control fixed visual conditional-to-place task with the same delay is not impaired, showing that it is recall after one-trial (or rapid, episodic) learning that is impaired (Kesner et al. 2008). CA3 NMDA receptors are, as predicted by the theory, necessary for rapid / one-trial spatial learning, as shown by a mouse knockout study by Nakazawa, Tonegawa and colleagues (Nakazawa et al. 2003, 2004; Tonegawa et al. 2003). As described above, we have shown that primate hippocampal CA3 neurons reflect the computational processes necessary for one-trial object-place event memory, used as a model for episodic memory (Rolls and Xiang 2006).

The theory predicts that, if primates including humans can form an episodic memory in which objects or people are seen at particular locations, even though the observer viewing the space has never been to those locations “out there” in space, a neural system in CA3 should exist that can support such associations between places “out there” within a scene and objects. Exactly this system is provided by the spatial view neurons that Rolls and colleagues discovered in CA3 (Georges-François et al. 1999; Robertson et al. 1998; Rolls et al. 1997a, 1998, 2005; Rolls and Xiang 2005, 2006). Place cells (Hartley et al. 2014; O’Keefe 1984; O’Keefe and Dostrovsky 1971) do not suffice for this type of episodic memory.

Another type of test of the autoassociation (or attractor) hypothesis for CA3 has been to train rats in various environments, e.g., a square and a circular environment and then test the prediction of the hypothesis that, when presented with an environment ambiguous between these, hippocampoal neurons will fall in an attractor state that represents one of the two previously learned environments but not a mixture of the two environments. Evidence consistent with the hypothesis has been found (Wills et al. 2005). In a particularly dramatic example, Jezek et al. (2011) discovered that, within each theta cycle, hippocampal pyramidal neurons may, in an ambiguous environment, represent one or other of the learned environments. This is an indication, predicted by Rolls and Treves (1998), that autoassociative memory recall can take place sufficiently rapidly to be complete within one theta cycle (120 ms) and that theta cycles might provide a mechanism for a fresh retrieval process to occur after a reset caused by the inhibitory part of each theta cycle. Thus, the memory can be updated rapidly to reflect a continuously changing environment and not remain too long in an attractor state.

### Recall via CA1 to neocortex

Tests of the theory reveal quantitatively and analytically the way that memories can be retrieved from the hippocampus to the neocortex (Treves and Rolls 1994). Memory retrieval has been shown, by the simulation of the multistage hippocampal system, including the entorhinal cortex, dentate, CA3 and CA1 and the return to the entorhinal cortex for recall, to be quantitatively realistic (Rolls 1995).

Many further tests of the theory are described elsewhere (Kesner et al. 2012; Kesner and Rolls 2015; Rolls 2016a).

## Final points

### The human hippocampus and the art of memory

The hippocampal processes described here for primates include recalling objects from spatial view recall cues. The theory has been developed that exactly this type of recall is involved in the “art of memory” used since classical times (Rolls 2017). Simonides of Ceos lived to tell the story of how, when a banquet hall collapsed in an earthquake, he could identify all the victims by recalling who had been sitting at each place at the table (Cicero 55 BC). This way of remembering items was developed into what has become known as *ars memoriae* by Roman senators who presented complex legal arguments in speeches that might last a whole day; they achieved this feat by associating each step in their argument with a location in a spatial scene through which their memory could progress from one end to the other during the speech, thus enabling them to recall each item in the correct order (Yates 1992). The procedure is also known as the “method of loci”. Phrases such as “in the first place” and “in the second place” probably refer to this method. Empirical work has demonstrated that the method of loci is efficacious (De Beni and Cornoldi 1985; Moe and De Beni 2005). Moreover, the activity of neurons in the human medial temporal lobe has been related to object-place memory and recall (Ison et al. 2015).

The new theory (Rolls 2017) is that this type of memory, *ars memoriae*, is implemented in the CA3 region of the hippocampus in which, in primates, spatial view cells can be found that allow a particular view to be associated with a particular object in an event or episodic memory. Given that the CA3 cells, with their extensive recurrent collateral system connecting different CA3 cells and with their associative synaptic modifiability, form an autoassociation or attractor network, the spatial view cells with their approximately Gaussian view fields become linked in a continuous attractor network. As the view space is traversed continuously (for example, by self-motion or imagined self-motion across the scene), the views are therefore successively recalled in the correct order, with no view missing and with low interference between the items to be recalled. Given that each spatial view has been associated with a different discrete item, the items are recalled in the correct order, with none missing. The theory provides a foundation for understanding the implementation of the key feature of *ars memoriae*, namely the ability to use a spatial scene to encode a sequence of items to be remembered (Rolls 2017).

### The sites of memory storage in the hippocampal system

A summary and clarification of where memories are stored in the hippocampal system and the roles of spatial representations in the theory described here might be helpful at this point. The theory is that the CA3 receives spatial and object information and can bring such information together by CA3-CA3 associative synaptic modification. Because this is a fast learning process, taking place in one trial, it is an unstructured memory about a particular event or episode and not a structured semantic memory. During storage, at least at one stage of the backprojection pathway to neocortex after CA3, associative learning between the backprojected information and the incoming information would occur to enable the correct neocortical representations in, for example, object or spatial cortical areas to be retrieved. To facilitate the latter retrieval, CA1 may then remap the separate parts of an event memory to a single representation (with the parts no longer separate for the whole memory) by using competitive learning in order later to provide an efficient recall cue (Kesner and Rolls 2015; Rolls 2016a). The dentate granule cells may operate as a competitive network to contribute to pattern separation before the CA3 cells and may use this mechanism to remap grid cells to place or spatial view cells. The connectivity from the dentate granule cells to the CA3 cells via the mossy fibres has a low probability of connectivity for contributing to pattern separation in CA3. Because the main function of the dentate to CA3 synapses is pattern separation and not information storage, these synapses are not associatively modifiable and, therefore, the neurogenesis of dentate granule cells can help pattern separation. This is the storage process.

Recall takes place in CA3 when a partial retrieval cue is applied, for example, the place, so that the whole memory is recalled by completion in the CA3 autoassociation or attractor network. The object information reaches the hippocampus from the inferior temporal visual cortex via the perirhinal and lateral entorhinal cortex. Reward information reaches the hippocampus from the orbitofrontal cortex and amygdala via the perirhinal and entorhinal cortex. Spatial information reaches the hippocampus from the parietal cortex (including the precuneus and also the posterior cingulate and retrosplenial cortex) via the parahippocampal gyrus (areas TF and TH) and medial entorhinal cortex. The entorhinal cortex grid cell system has multiple attractors that perform idiothetic update (path integration) in the dark. In rodents, the spatial information is primarily about the place in which the rodent is located. In primates, the information is about spatial view, with probably some modulation by place. The difference from rodents is that primates have a fovea and, hence, the high resolution view of a small part of the environment results in the object that is being fixated forming the spatial input (de Araujo et al. 2001; E.T. Rolls and S. Wirth in preparation). Spatial scene information may also reach the primate hippocampus from the temporal cortex scene area (Kornblith et al. 2013). The CA3 system is not suitable for a continuous attractor for spatial navigation, because the object information would make the continuous attractor too bumpy to work well (Cerasti and Treves 2013). Path integration for this reason is performed in the medial entorhinal cortex. The CA3 however is able to combine continuous spatial with discrete object representations and to recall the complete representation from either a spatial or object cue (Rolls et al. 2002).

## General concluding remarks

In conclusion, a theory of hippocampal function has been described. This goes beyond a model by incorporating many analytic results concerning, for example (1) the importance of the number of synapses onto each CA3 neuron and the sparseness of the representation for providing an estimate of the memory capacity of the hippocampus, (2) the distinct roles of the mossy fibre and perforant path inputs to the CA3 neurons and (3) the way that information of recently learned episodic information can be recalled to the neocortex from the hippocampus by using the multistage cortico-cortical backprojection pathway (Rolls 2010, 2016a; Treves and Rolls 1992, 1994). For recall, the hippocampal output can be thought of as a pointer to neocortical neurons via which memories can be called by using the backprojection pathways (Kesner and Rolls 2015; Rolls 2016a). The approach underlines the importance of understanding spatial representations in the primate, including the hippocampus, because they are different from those in rodents and are relevant to understanding episodic memory in humans in which memories of where objects and rewards are within an environment can be formed by an individual, without that individual ever having been present in the place. The difference may be related to the great importance and development of vision in primates; this has also had implications in our understanding of the cortical organization of many other processing systems, including those involved in taste processing and in emotion (Rolls 2014). In primates, extensive development has occurred of many neocortical areas, in part related to the great expansion of cortical visual computation (Rolls 2012c) and these cortical developments are also important in understanding hippocampal function and its relationship to episodic memory and to spatial processing (E.T. Rolls and S. Wirth in preparation). In addition, the approach has emphasized the importance of founding the theory on details of anatomy, quantitatively where possible and of the neurophysiology (and I include functional neuroimaging) of the brain systems involved.

## Electronic supplementary material

ESM 1 (DOCX 88 kb)
