# Supplementary material for: The storage and recall of memories in the hippocampo-cortical system
Source: Cell Tissue Res. 2017 Dec 7;373(3):577–604. doi: 10.1007/s00441-017-2744-3 (PMC6132650; doi:10.1007/s00441-017-2744-3)
Supplement: Supplementary file 1 — (DOCX 88 kb) [file 441_2017_2744_MOESM1_ESM.docx]

**The storage and recall of memories in the hippocampo-cortical system**

**Supplementary material**

Edmund T Rolls

Oxford Centre for Computational Neuroscience, Oxford, England

and University of Warwick, Department of Computer Science, Coventry, England.

Correspondence: Email: [Edmund.Rolls@oxcns.org](mailto:Edmund.Rolls@oxcns.org). Url: http://www.oxcns.org

In Cell and Tissue Research (2018) Special Issue on Hippocampal structure and function, guest-edited by Andreas Draguhn, Oliver von Bohlen und Halbach and Jon Storm-Mathisen

**Box 1. Summary of the architecture and operation of autoassociation or attractor networks**

The prototypical architecture of an autoassociation memory is shown in Fig. S1. The external input *e_i_* is applied to each neuron *i* by unmodifiable synapses. This produces firing *y_i_* of each neuron. Each neuron is connected by a recurrent collateral synaptic connection to the other neurons in the network, via associatively modifiable connection weights *w_ij_*. This architecture effectively enables the vector of output firing rates to be associated during learning with itself. Later on, during recall, presentation of part of the external input will force some of the output neurons to fire, but through the recurrent collateral axons and the modified synapses, other neurons can be brought into activity. This process can be repeated a number of times, and recall of a complete pattern may be perfect. Effectively, a pattern can be recalled or recognized because of associations formed between its parts. This of course requires distributed representations.


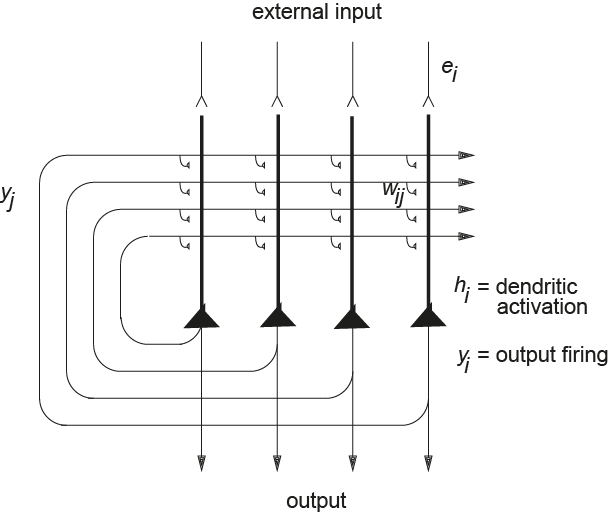


**Fig. S1** The architecture of an autoassociative neural network. The recurrent collateral synaptic weights are excitatory. Inhibitory neurons (not illustrated) must be present to control the firing rates.

Training for each desired pattern occurs in a single trial. The firing of every output neuron *i* is forced to a value *yi* determined by the external input *ei* Then a Hebb-like associative local learning rule is applied to the recurrent synapses in the network:

δ*w_ij_* = *k* . *y_i_* . *y_j_* (1)

where *k* is a learning rate constant, *y_i_* is the activation of the dendrite (the postsynaptic term), *y_j_* is the presynaptic firing rate, and δ*w_ij_* is the change in the synaptic weight from axon *j* to neuron *i*. This learning algorithm is fast, ‘one-shot’, in that a single presentation of an input pattern is all that is needed to store that pattern.

It is notable that in a fully connected network, this will result in a symmetric matrix of synaptic weights, that is, the strength of the connection from neuron 1 to neuron 2 will be the same as the strength of the connection from neuron 2 to neuron 1 (both implemented via recurrent collateral synapses).

During recall, an external input *e* is applied, and produces output firing, operating through a non-linear activation function. The firing is fed back by the recurrent collateral axons shown in Fig. 3 to produce activation of each output neuron through the modified synapses on each output neuron. The activation *h_i_* produced by the recurrent collateral effect on the *i*th neuron is, in the standard way, the sum of the activations produced in proportion to the firing rate of each axon operating through each modified synapses *w_ij_*, that is,

*h_i_* = ∑*_j_ y_j_* *w_ij_* (2)

where the sum is over the input axons to each neuron, indexed by *j*.

The output firing *y_i_* is a function of the activation produced by the recurrent collateral effect (internal recall) and by the external input *e_i_* :

*y_i_* = f(*h_i_* + *e_i_*) (3)

The activation function f should be non-linear, and may be for example binary threshold, linear threshold, sigmoid, etc. A purely linear system would not produce any categorization of the input patterns it receives, and therefore would not be able to effect anything more than a trivial (i.e. linear) form of completion and generalization.

During recall, a part of one of the originally learned stimuli can be presented as an external input. The resulting firing is allowed to iterate repeatedly round the recurrent collateral system, gradually on each iteration recalling more and more of the originally learned pattern. Completion thus occurs. If a pattern is presented during recall that is similar to one of the previously learned patterns, then the network settles into a stable recall state in which the firing corresponds to that of the previously learned pattern. The network can thus generalize in its recall to the most similar previously learned pattern. Important results (cf. Section 3.3.2) characterize how many patterns can be stored in a network in this way without interference during recall.

Details are provided by Rolls (2008, 2016) in Appendices B.

**Box 2. Summary of the architecture and operation of pattern association networks**


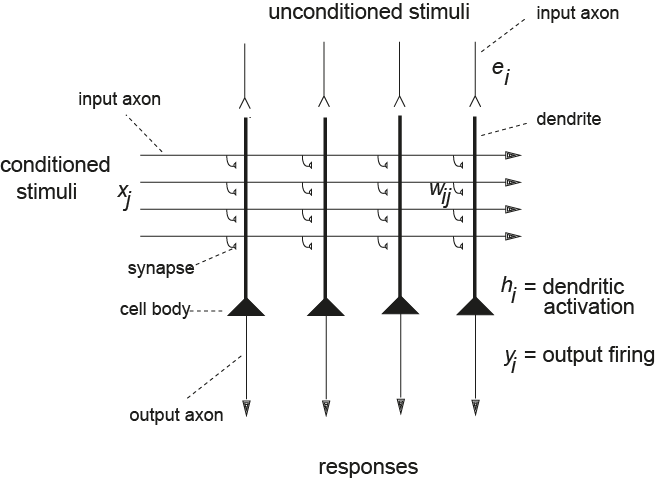


Fig. S2. The architecture of a pattern association network. An unconditioned stimulus (US) has activity or firing rate *e_i_* for the *i’*th neuron, and produces firing *y_i_* of the *i’*th neuron which is an unconditioned response (UR). The conditioned stimuli (CS) have activity or firing rate *x_j_* for the *j’*th axon. During learning, a CS is presented at the same time as a US, and the synaptic weights are modified by an associative synaptic learning rule δ*w_ij_* = *k* . *y_i_* . *x_j_* where *k* is a learning rate constant, and δ*w_ij_* is the change in the synaptic weight from axon *j* to neuron *i*. During recall, only the CS is presented, and the activation *h_i_* is calculated as a dot product between the input stimulus and the synaptic weight vector on a neuron *h_i_* = ∑*_j_ x_j_* *w_ij_* where the sum is over the input axons to each neuron, indexed by *j*. The output firing *y_i_* is a function f of the activation *y_i_* = f(*h_i_*). The activation function f should be non-linear, and may be for example binary threshold, linear threshold, or sigmoid. Inhibitory neurons not shown in the figure are part of the way in which the threshold of the activation function is set. The non-linearity in the activation function enables interference from other pattern pairs stored in the network to be minimized. The pattern association network thus enables a CS to retrieve a response, the conditioned response (CR), that was present as a UR during the learning. An important property is that if a distorted CS is presented, generalization occurs to the effects produced by the closest CS during training. Details are provided by Rolls (2008, 2016) in Appendices B.

**Box 3. Summary of the architecture and operation of competitive networks**


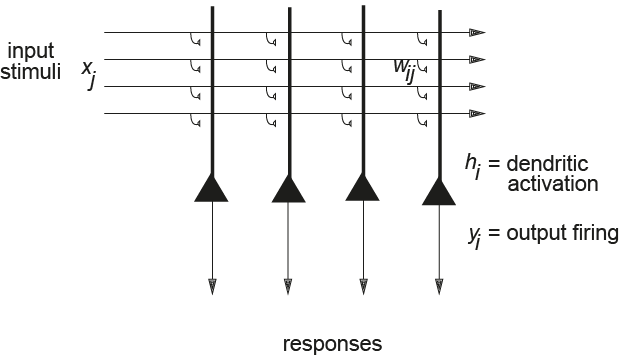


Fig. S3. The architecture of a competitive network. During training, an input stimulus is presented to the synaptic matrix which has random initial weights *w_ij_* and produces activation *h_i_* of the *i’*th neuron calculated as a dot product between the input stimulus and the synaptic weight vector on a neuron *h_i_* = ∑*_j_ x_j_* *w_ij_* where the sum is over the input axons to each neuron, indexed by *j*. The output firing *y_i_* is a function f of the activation *y_i_* = f(*h_i_*). The activation function f should be non-linear, and may be for example binary threshold, linear threshold, or sigmoid. Inhibitory neurons not shown in the figure are part of the way in which the threshold of the activation function is set in which the threshold reflects the firing of all the output neurons. This or other competitive mechanisms result in a typically sparse set of output neurons having firing after the competition.

Next an associative synaptic modification rule is applied, while the presynaptic input and the postsynaptic output are both present, δ*w_ij_* = *k* . *y_i_* . *x_j_* where *k* is a learning rate constant, and δ*w_ij_* is the change in the synaptic weight from axon *j* to neuron *i*.

Next the synaptic weight vector on each neuron is normalized, to ensure that no one neuron dominates the classification.

This process is repeated for every input pattern in random permuted sequence, and this process is repeated for a small number of training epochs.

After the training, each dendritic weight vector points towards a cluster of patterns in the input space. The competitive network has used self-organization with no teacher to categorize the inputs, with patterns close in the input space activating the same set of output neurons, and different clusters of inputs activating different sets of output neurons.

Details are provided by Rolls (2008, 2016) in Appendices B.

References

Rolls ET (2008) Memory, Attention, and Decision-Making: A Unifying Computational Neuroscience Approach. Oxford University Press, Oxford

Rolls ET (2016) Cerebral Cortex: Principles of Operation. Oxford University Press, Oxford
